# Manipulation of PD‐L1 Endosomal Trafficking Promotes Anticancer Immunity

**DOI:** 10.1002/advs.202206411

**Published:** 2022-12-25

**Authors:** Zuodong Ye, Yiding Xiong, Wang Peng, Wenjie Wei, Lihong Huang, Juliana Yue, Chunyuan Zhang, Ge Lin, Feng Huang, Liang Zhang, Songguo Zheng, Jianbo Yue

**Affiliations:** ^1^ City University of Hong Kong Shenzhen Research Institute Shenzhen 518057 China; ^2^ Department of Biomedical Sciences City University of Hong Kong Hong Kong 999077 China; ^3^ Department of Clinical Immunology Third Affiliated hospital at the Sun Yat‐sen University Guangzhou 510630 China; ^4^ Research Core Facilities South University of Science and Technology of China Shenzhen 518052 China; ^5^ Department of Biology Brigham Young University Provo UT 84602 USA; ^6^ School of Biomedical Sciences The Chinese University of Hong Kong Hong Kong 999077 China; ^7^ Division of Natural and Applied Sciences Synear Molecular Biology Lab Duke Kunshan University Kunshan 215316 China

**Keywords:** 6J1, anticancer immunity, endocytosis, endosomal trafficking, extracellular vesicle, PD‐L1, Rab5, Rab27

## Abstract

The aberrant regulation of PD‐L1 in tumor cells remains poorly understood. Here, the authors systematically investigate the endosomal trafficking of plasma membrane PD‐L1 in tumor cells. They show that plasma membrane PD‐L1 is continuously internalized, and then trafficked from early endosomes to multivesicular bodies/late endosomes, recycling endosomes, lysosomes, and/or extracellular vesicles (EVs). This constitutive endocytic trafficking of PD‐L1 is Rab5‐ and clathrin‐dependent. Triazine compound 6J1 blocks the endosomal trafficking of PD‐L1 and induces its accumulation in endocytic vesicles by activating Rab5. 6J1 also promotes exosomal PD‐L1 secretion by activating Rab27. Together, these effects result in a decrease in the membrane level of PD‐L1 in 6J1‐treated tumor cells and enables tumor cells to be more susceptible to the tumor‐killing activity of T cells in vitro. 6J1 also increases tumor‐infiltrating cytotoxic T cells and promotes chemokines secretion in the tumor microenvironment. Rab27 knockdown abolishes 6J1‐induced PD‐L1 secretion in EVs and revokes the exhausted tumor‐infiltrating T cells in tumors, thereby improving the anticancer efficacy of 6J1. Furthermore, a combination of 6J1 and an anti‐PD‐1 antibody significantly improves the anticancer immune response. Therefore, manipulating PD‐L1 endosomal trafficking provides a promising means to promote an anticancer immune response in addition to the immune checkpoint‐blocking antibody therapy.

## Graphical abstract

The plasma membrane PD‐L1 undergoes a constitutive endosomal trafficking process. 6J1 (a triazine compound) blocks PD‐L1's endosomal trafficking and induces its secretion in extracellular vesicles (EVs), decreasing PD‐L1 at the plasma membrane. A combination of 6J1 with the inhibition of PD‐L1 secretion in EVs or with a PD‐1 monoclonal antibody significantly promotes the anticancer immune response.

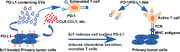

## 1 Introduction

The T cell‐mediated immune system can be harnessed to control tumor cell growth. The protein called programmed death‐1 (PD‐1, also known as CD279), is one of the critical immune checkpoint proteins in activated T cells. It interacts with its ligand, PD‐L1 (also called CD274 or B7‐H1), in tumor cells or other antigen‐presenting cells (APCs). This interaction inhibits the activation and expansion of cytotoxic T cells and enables tumor cells to escape immune surveillance.^[^ ^1^ ^]^ It has been shown that PD‐L1 is overexpressed or dysregulated in various tumor cells.^[^ ^2^ ^]^ Disruption of the PD‐L1/PD‐1 axis using blocking antibodies against these checkpoint proteins has significant clinical benefits and durable responses in different cancer types.^[^ ^3^ ^]^ Indeed, the success of immune checkpoint inhibitors (ICIs) has revolutionized cancer immunotherapy in recent years.^[^ ^4^ ^]^ For example, several PD‐L1 or PD‐1 antibodies, such as Nivolumab (Opdivo) and Durvalumab (Imfinzi), have already been approved by the Food and Drug Administration (FDA) and European Medicines Agency (EMA). However, only a subset of the patients treated with these drugs actually finds them beneficial, due to the development of drug resistance either during or after ICI treatment.^[^ ^5^ ^]^ Furthermore, the relatively large size of antibody drugs might limit their therapeutic efficacy due to their relatively low ability to penetrate into the complex tumor microenvironment (TME).^[^ ^6^ ^]^

PD‐L1 is subjected to transcriptional, posttranscriptional, translational, and posttranslational regulation by multiple signaling pathways.^[^ ^7^ ^]^ The correlation between its expression and the efficacy of ICIs in tumor cells is highly context‐dependent, for example, regarding the tumor cell type and the specific TME. For example, in melanoma cells and colon cancer cells, loss‐of‐function mutations of Janus Kinase 1/2 (JAK1/2) led to a decreased level of PD‐L1, which resulted in the cells becoming resistant to PD1 blockade therapy.^[^ ^8^ ^]^ On the other hand, oncogenic pathways (such as phosphoinositide 3‐kinases (PI3K) and signal transducer and activator of transcription 3 (STAT3)), have been shown to upregulate the expression of PD‐L1 on tumor cells. This might, therefore, partially restore PD‐L1/PD‐1 function by providing more PD‐L1 binding sites to impair the neutralization efficiency of the antibodies.^[^ ^9^ ^]^ However, the mechanism by which PD‐L1 is dysregulated in tumor cells to evade immune surveillance, especially with regard to its intracellular trafficking, remains elusive. Understanding the mechanisms of PD‐L1 regulation in tumor cells and developing effective approaches to target PD‐L1 are essential for improving the therapeutic efficacy of ICIs and overcoming drug resistance.

PD‐L1 is a type I transmembrane protein, which undergoes endosomal trafficking in tumor cells. It has been reported that CKLF‐like MARVEL transmembrane domain containing 4 (CMTM4) and CMTM6 direct the internalized PD‐L1 to recycling endosomes, and in this way, they protect it from being delivered to lysosomes for degradation.^[^ ^10^ ^]^ The inhibition of endocytic recycling has been shown to downregulate the level of membrane PD‐L1.^[^ ^10a^ ^]^ However, details about the mechanism involved in the endosomal trafficking of PD‐L1 remain elusive.

Following an early high‐content phenotypic screen that identified several triazine compounds, for example, vacuolin‐1 (V1), as exocytosis inhibitors,^[^ ^11^ ^]^ we identified V1 or its analogs as inhibitors of endosomal trafficking and autophagy. We further demonstrated that V1 targets CapZ (a stable heterodimeric protein complex consisting of *α* and *β* subunit), to over‐activate Rab5, thus inhibiting the early‐to‐late endosome transition. V1 inhibited metastasis but not tumor growth in nude mice.^[^ ^12^ ^]^

Here, we showed that PD‐L1 at the plasma membrane was continuously internalized via endocytosis, and once internalized, PD‐L1 colocalized with Rab5‐positive early endosomes, before being directed to recycling compartments, multivesicular bodies (MVBs), lysosomes, and/or secretory vesicles. We synthesized a series of V1 analogs and identified 6J1 as being one of the most potent agents to block endosomal trafficking. We showed that 6J1 locked PD‐L1 onto early endosomes by activating Rab5, which prevented it from being trafficked to lysosomes or recycling endosomes. This inhibitor also increased the exosomal secretion of PD‐L1 by activating Rab27. Together, these effects led to a decrease in the level of membrane PD‐L1. The combination of 6J1 with inhibition of PD‐L1's exosomal secretion or anti‐PD‐1 antibody significantly improved the antitumor immune response.

## 2 Results

### 2.1 The Endosomal Trafficking of Membrane PD‐L1 is Clathrin‐ and Rab5‐Dependent

**Figure 1:**
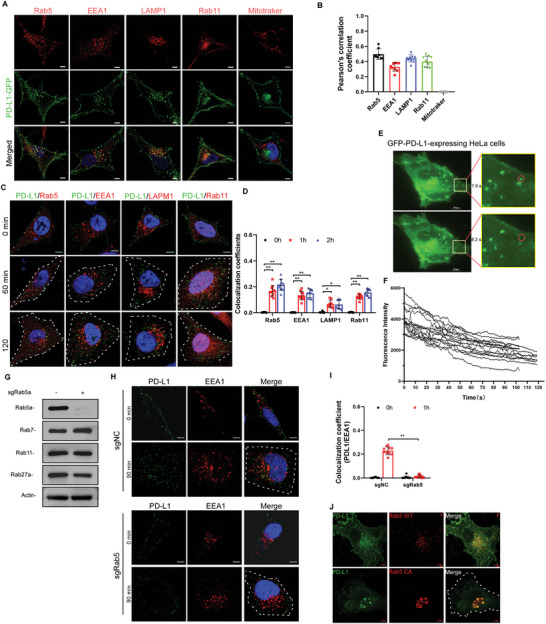
Plasma membrane PD‐L1 traffics through Rab5‐dependent endocytotic pathway. A) PD‐L1‐GFP expressing HeLa cells were stained with antibodies (red) against Rab5, EEA1, LAMP1, Rab11, or with mitotracker deep red probe. Scale bar is 5 µm. B) Pearson's correlation coefficient (PCC) analysis of images in (A). C) HeLa cells were plated on coverslips in 24‐well plates and incubated with the anti‐PD‐L1 antibody on ice for 60 min. Thereafter, the cells were incubated with warm medium at 37 °C for the indicated time points, and the cells were then fixed and stained with antibodies (red) against Rab5, EEA1, LAMP1, or Rab11. Scale bar is 5 µm. D) Manders colocalization coefficient (MCC) analysis of images in (C). E,F) Representative live‐cell image of PD‐L1‐GFP expressing HeLa cell acquired by TIRF at two time points (left). The green box is zoomed in on the right (E). The intensity of various PD‐L1 puncta in the cell was tracked through time course and quantified (F). The decrease in PD‐L1 puncta intensity represents the internalization of PD‐L1 from cell surface to cytoplasm. G) Western blot analysis of the knockdown efficiency of Rab5. H) Rab5‐knockdown HeLa cells were stained with the anti‐PD‐L1 antibody (green) on ice for 1 h. 1.5 h after being incubated at a warm medium at 37 °C, the cells were fixed and stained with antibodies against EEA1 (red). The scale bar is 5 µm. I) MCC analysis of images in (H). J) PD‐L1‐GFP‐expressing HeLa cells were transfected with Rab5‐WT or Rab5‐CA (constitutively active Rab5Q79L). Cells were then fixed and imaged. Scale bar is 5 µm. The graphs represented data from three independent experiments, and data quantifications were expressed as mean ± s.e.m. *P < 0.05, **P < 0.01, ***P < 0.001. MCC, Manders colocalization coefficient; PCC, Pearson's correlation coefficient.

The endosomal trafficking of PD‐L1 remains poorly studied thus far. In live GFP‐PD‐L1‐expressing HeLa cells, GFP‐PD‐L1 puncta were detected both at the plasma membrane and in the cytoplasm. The cytosolic GFP‐PD‐L1 puncta exhibited strong colocalization with Rab5 (an early endosome marker), EEA1 (an early endosome marker), LAMP1 (a late endosome/lysosome marker), Rab11 (a recycling endosome marker), but not with MitoTracker (a mitochondria probe) (**Figure** **1**A, 1B). Similar results were observed in fixed cells that were double‐immunolabeled with an anti‐PD‐L1 antibody and antibodies against the aforementioned proteins (Figure S1A, Supporting Information). These results suggest that PD‐L1 in the plasma membrane might be subjected to an active endosomal trafficking process. To monitor the endosomal trafficking of PD‐L1, live HeLa cells were labeled with an anti‐PD‐L1 antibody on ice for 1 h, and then they were either fixed immediately or else they were incubated at 37 °C for 60 min or 120 min to initiate the internalization of the PD‐L1‐antibody complex prior to fixation. The fixed cells were then immunolabeled with antibodies to Rab5, EEA1, LAMP1, or Rab11. We showed that the internalized PD‐L1 exhibited strong colocalization with these endocytic vesicle markers (Figure 1C, 1D). We further monitored the trafficking of plasma membrane PD‐L1 in live GFP‐PD‐L1‐expressing cells by TIRF microscopy. In TIRF imaging, when the fluorescence labeling membrane protein leaves the plasma membrane, the fluorescent intensity will decrease or lose depending on the distance of protein moving away from the plasma membrane. Indeed, when membrane PD‐L1 (showing as green puncta) moved away from the plasma membrane, the fluorescence intensity of PD‐L1 puncta gradually decreased even in the absence of antibody or ligand binding (Figure 1E, 1F, and Video S1, Supporting Information). These results indicate that plasma membrane PD‐L1 is subjected to continuous endocytic trafficking.

We subsequently set out to determine the molecular mechanisms underlying PD‐L1's endosomal trafficking. First, we treated cells with or without Pitstop2 (an inhibitor of clathrin‐dependent endocytosis),^[^ ^13^ ^]^ or Filipin III (an inhibitor of caveolae‐mediated endocytosis),^[^ ^14^ ^]^ and then incubated the control or treated cells with PD‐L1 antibody on ice. The labeled cells were subsequently kept in the warm medium for 60 min to trigger the endocytosis of the PD‐L1‐antibody complex, followed by fixation and immunostaining with an anti‐Rab5 antibody. We showed that in control or Filipin III‐treated cells, PD‐L1 was endocytosed normally, manifested by the strong colocalization between PD‐L1 and Rab5, whereas in Pitstop2‐treated cells, the endocytosis of PD‐L1 was blocked, manifested by the poor colocalization between PD‐L1 and Rab5 (Figure S1B, Supporting Information). These results suggest that PD‐L1's endocytosis is clathrin‐dependent.

Next, we knocked down the expression of Rab5a by CRISPR‐Cas9 in HeLa cells (Figure 1G) and then assessed the endosomal trafficking of PD‐L1 in control or Rab5‐knockdown cells. The trafficking of the membrane PD‐L1 was examined by its colocalization with EEA1 and Rab11. We showed that in Rab5‐knockdown cells, membrane PD‐L1 was internalized, but stayed close to the plasma membrane and exhibited poor colocalization with EEA1 and Rab11 when compared to control cells (Figure 1H, 1I, Figure S1C, S1D, Supporting Information). On the other hand, overexpression of a constitutively‐active mutant of Rab5 (Rab5 CA, Rab5^Q79L^),^[^ ^15^ ^]^ locked PD‐L1 in enlarged endosomes (Figure 1J). These results suggest that the endosomal trafficking of PD‐L1 is Rab5 dependent. As a control, we knocked down Rab27a by shRNAs in HeLa cells (Figure S1E, Supporting Information); this is required for the fusion of MVBs with the plasma membrane for extracellular vesicle (EVs) secretion.^[^ ^16^ ^]^ We showed that Rab27a knockdown had little effect on the colocalization between internalized PD‐L1 and CD63 (an MVB/late endosomal marker) (Figure S1F, Supporting Information), suggesting that the trafficking of membrane PD‐L1 to early endosome or MVB is Rab27‐independent. In summary, these data suggest that plasma membrane PD‐L1 is continuously internalized, and when it is being carried by endocytic vesicles, it is trafficked from early endosomes to either MVBs/late endosomes, or recycling endosomes in a Rab5‐dependent manner.

### 2.2 The Extracellular Vesicle Secretion of PD‐L1 is Dependent on Rab5 and Rab27 but Autophagy‐Independent

**Figure 2:**
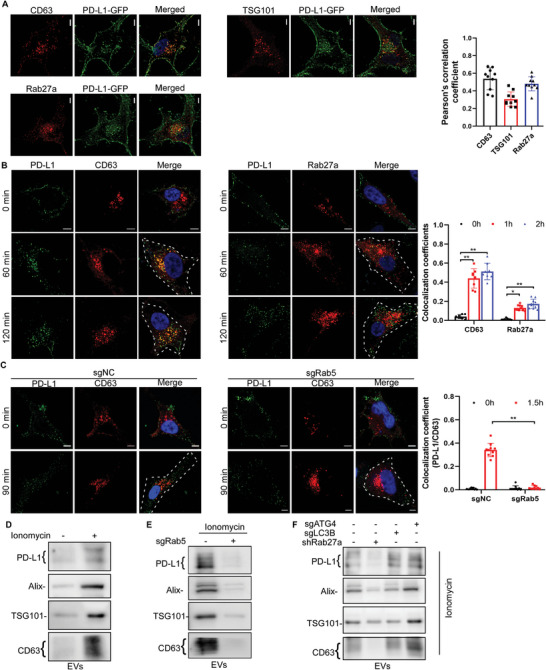
The secretion of PD‐L1 in EVs is dependent on Rab27 and Rab5 but autophagy‐independent. A) PD‐L1‐GFP expressing HeLa cells were transfected with CD63‐mcherry, TSG101‐mcherry, or Rab27a‐mcherry, after which they were fixed and imaged. PCCs of PD‐L1 with CD63, TSG101, or Rab27 were quantified. B) HeLa cells were plated on coverslips in 24‐well plates and incubated on ice with the anti‐PD‐L1 antibody (green) for 60 min, after which some were fixed immediately whereas others were incubated with medium at 37 °C for 60 or 120 min prior to fixation. All the fixed cells were then immunolabeled with antibodies against CD63 or Rab27a (red). MCCs of PD‐L1 with CD63 or Rab27 were quantified. C) Rab5‐knockout HeLa cells were transfected with CD63‐mcherry and incubated on ice with the anti‐PD‐L1 antibody (green) for 60 min. Some cells were fixed immediately whereas others were incubated with medium at 37 °C for 90 min before fixation. MCCs of PD‐L1 with CD63 were quantified. D,E) HeLa cells (D) or Rab5‐knockout HeLa cells (E) were treated with or without ionomycin (1 µm) for 48 h, and then the EVs were purified from the supernatant and subjected to western blot analysis. F) EVs were purified from the supernatant of Rab27A‐, LC3B‐, or ATG4B‐knockdown cells, and subjected to western blot analysis. In (A–C), the scale bars are 5 µm, and the bar graphs represent mean ± s.e.m. of three independent experiments. The asterisks indicate significant differences at *P < 0.05 and ** *P* < 0.01. “ns” indicates data that are not significantly different.

PD‐L1 has previously been shown to be secreted out of tumor cells via EVs.^[^ ^17^ ^]^ We showed that the cytosolic PD‐L1‐GFP puncta indeed exhibited strong colocalization with various MVBs or EV markers (e.g., CD63, TSG101, and Rab27a), in PD‐L1‐GFP‐expressing cells (**Figure** **2**A). Similar results were observed in fixed cells co‐labeled with an anti‐PD‐L1 antibody and antibodies against CD63 or Rab27a (Figure S2A, S2B, Supporting Information). We then examined whether some of the endocytosed membrane PD‐L1 is routed to EVs for secretion. Indeed, after membrane PD‐L1 was internalized, it exhibited strong colocalization with CD63 and RAB27 (Figure 2B). Moreover, Rab5 knockdown abolished the colocalization between internalized PD‐L1 and CD63 (Figure 2C). PD‐L1 was detected in the EVs, and ionomycin (a calcium ionophore), which induces EVs secretion,^[^ ^18^ ^]^ markedly increased the PD‐L1 level in EVs (Figure 2D). As expected, Rab5 knockdown abolished the ionomycin‐induced increase of PD‐L1 (and other proteins) in EVs (Figure 2E). These results indicate that after endocytosis, some membrane PD‐L1 goes to EVs for secretion in a Rab5‐dependent manner.

The amphisome, which is formed when an autophagosome fuses with an MVB/late endosome, has been implicated in the secretion of EVs.^[^ ^19^ ^]^ Since we found that PD‐L1 was associated with LC3B (Figure S2C, Supporting Information), it is possible that the canonical autophagic machinery might be involved in PD‐L1 secretion. However, LC3B or ATG4B knockdown (Figure S2D, S2E, Supporting Information) did not affect PD‐L1 levels in EVs (Figure 2F). Bafilomycin A1 (Baf A1), an inhibitor of the vacuolar H^+^ ATPase, blocks the fusion of lysosomes with autophagosomes or late endosomes.^[^ ^20^ ^]^ Interestingly, although Baf A1 markedly increased both the total and EVs levels of PD‐L1 in cells, Torin1, an autophagy activator, had little effect on either of these levels (Figure S2F, S2G, Supporting Information). Furthermore, Rab27 was found to function in MVB and regulate the exosomal secretion process.^[^ ^21^ ^]^ As expected, Rab27a knockdown significantly decreased the PD‐L1 levels in EVs (Figure 2F). These results suggest that the EVs secretion of PD‐L1 is Rab27‐dependent but independent of the canonical autophagy machinery.

### 2.3 6J1, A Triazine Compound, Potently Compromises the Endosomal Trafficking of PD‐L1

Since plasma membrane PD‐L1 is subjected to constitutive endosomal trafficking (Figures 1 and 2, and Figures S1 and S2, Supporting Information), we speculated that compromising this process might change the levels of PD‐L1 at the cell surface, and in this way, it might affect the anticancer immune response in vivo. We have previously identified several triazine compounds, for example, V1, as inhibitors of endosomal trafficking and autophagy.^[^ ^12a,b,d^ ^]^ To improve the efficacy and solubility of these triazine compounds, we synthesized another 21 triazine derivatives (Figure S3, Supporting Information), and assessed their ability to inhibit the endosomal trafficking of DQ‐BSA. DQ‐BSA is a self‐quenching dye, which enters cells via endocytosis and is then delivered to the lysosome for degradation; this relieves fluorescence‐quenching, resulting in the cells generating bright fluorescence.^[^ ^22^ ^]^ Several triazine derivatives, including 6J1, C3, C4, D3, D4, H1, and P2, potently inhibited the fluorescence intensity of DQ‐BSA‐labeled cells (Figure S4A, Supporting Information). We also tested whether these hit compounds could inhibit the recycling endosomal pathway by performing a Transferrin recycling assay. In this assay, the fluorescence intensity of Transferrin‐488‐labeled cells reflects the activity level of the recycling endocytic trafficking process. We showed that compared to TM201636 (a PIKfyve and known endocytic inhibitor), which acts as a positive control, all of the triazine compounds effectively inhibited Transferrin recycling (Figure S4B, Supporting Information).

**Figure 3:**
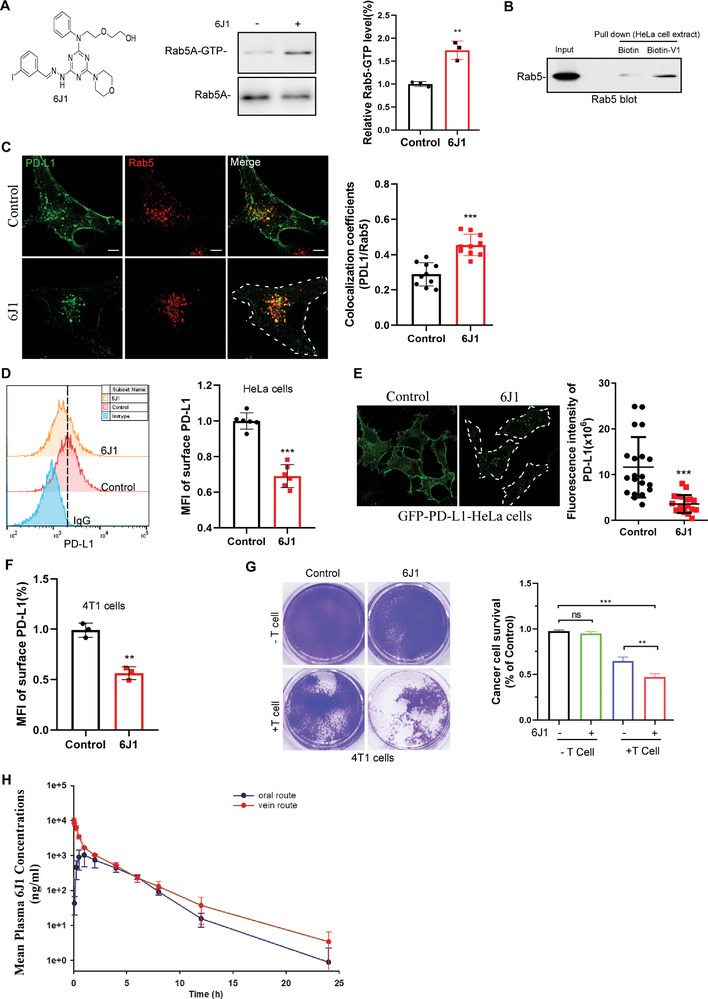
6J1 compromises the endosomal trafficking of PD‐L1. A) The level of active RAB5‐GTP in control or 6J1 (1 µm)‐treated HeLa cells was examined with a GST–R5BD pulldown assay. B) Lysates prepared from HeLa cells treated with biotin or biotin‐V1 were incubated with streptavidin beads, and then streptavidin pulldowns were subjected to Rab5 immunoblotting. C) PD‐L1‐GFP expressing HeLa cells were treated with or without 6J1 (1 µm) for 24 h. Cells were then fixed and immunolabeled with antibodies against Rab5 (red). MCCs of PD‐L1 with Rab5 were quantified. D) Flow cytometry‐based quantification of the plasma membrane levels of PD‐L1 in HeLa cells treated with or without 6J1 (1 µm) for 24 h. The mean fluorescence intensity of PD‐L1 was quantified. E) Confocal imaging of PD‐L1‐GFP‐expressing HeLa cells treated with or without 6J1 (1 µm) for 24 h. F) Flow cytometry‐based quantification of the plasma membrane levels of PD‐L1 in 4T1 cells treated with or without 6J1 (1 µm) for 24 h; the mean fluorescence intensity of PD‐L1 was quantified. G) T‐cell‐meditated tumor cell‐killing assay in 4T1 cells treated with or without 6J1 (1 µm). Activated T cells and 4T1 cells were cocultured in 12‐well plates for 3 days, and the surviving tumor cells were visualized using crystal violet staining. The relative fold ratios of crystal violet intensities are shown as a percentage of the control. H) Pharmacokinetic (PK) analysis of 6J1 (15 mg kg^−1^) via oral or tail‐vein delivery in rats. In (C, E), scale bars are 5 µm. In (A, C–G), the graphs represent mean ± s.e.m of three independent experiments, and the asterisks indicate significant differences at *P < 0.05, **P < 0.01, or ***P < 0.001. “ns” indicates data that are not significantly different.

Since 6J1 inhibited endosomal trafficking more potently and has higher polarity than the other triazine compounds (Figures S3 and S4A–S4C, Supporting Information), we characterized this compound in further detail. First, we examined the effects of 6J1 on the endosomal trafficking of EGFR. We showed that when 6J1‐pretreated HeLa cells were incubated with EGF, then the degradation of EGFR was inhibited (manifested by EGFR complex being locked on the endosomes). In comparison, the EGFR was completely degraded after EGF treatment in control cells (not pre‐treated with 6J1) (Figure S4D, Supporting Information). The phenotype of 6J1‐treated HeLa cells is reminiscent of that of Rab5 CA (RAB5^Q78L^) cells with regards to the homotypic fusion of early endosomes, and the early‐to‐late endosome transition being blocked.^[^ ^15^ ^]^ Thus, we performed a GST‐R5BD pulldown assay to detect the level of GTP‐bound Rab5,^[^ ^23^ ^]^ in cells treated with or without 6J1, and showed that 6J1 significantly increased the level of Rab5‐GTP (**Figure** **3**A). Biotin‐V1 (a 6J1 analog), also directly pulled down Rab5 from HeLa cell lysates (Figure 3B). Collectively, these results suggest that 6J1 arrests endosomal trafficking by activating Rab5.

We also tested the cytotoxicity of 6J1 in several cancer cell lines. We showed that 6J1 induced cell death and inhibited cell proliferation in a concentration‐dependent manner (Figure S4E, S4F, Supporting Information). Yet, 6J1 at its effective concentrations (i.e., ≤1 µm) hardly induced any cell death, and also only subtly inhibited cell proliferation in several cell types (Figure S4E, S4F, Supporting Information). Of note, 6J1 at 1 µm inhibited, not blocked, the endosomal trafficking (Figure S4A–S4D, Supporting Information). Therefore, these results suggest that cells tolerate the decreased endosomal trafficking processes.

Next, we examined whether 6J1 inhibits endosomal trafficking of PD‐L1. We showed that PD‐L1 accumulated in vesicle‐like structures in the cytoplasm of 6J1‐treated cells, and these vesicles exhibited strong colocalization with the early endosome markers, EEA1 and Rab5 (Figure 3C, Figure S4G, Supporting Information). Interestingly, the level of PD‐L1 at the cell surface of 6J1‐treated cells seemed to be lower than that of control cells (Figure 3C, Figure S4G, Supporting Information). Thus, we further quantified the levels of cell surface PD‐L1 in live cells treated with or without 6J1 both by flow cytometry and confocal imaging. We showed that indeed, 6J1 significantly reduced the levels of PD‐L1 at the plasma membrane in various tumor cells from either cancer cell lines (e.g., HeLa, 4T1, B16‐F10, and MDA‐MB‐231 cells) (Figure 3D–F, Figure S4H, S4I, Supporting Information). However, it did not inhibit the level of PD‐L1 mRNA in cells (Figure S4J, Supporting Information), suggesting that the 6J1‐induced decrease in cell surface PD‐L1 protein is not due to transcriptional inhibition. These results, therefore, suggest that the reduction of plasma membrane PD‐L1 observed in 6J1‐treated cells is likely due to an accumulation of internalized PD‐L1 in endocytic vesicles.

### 2.4 6J1 Suppresses the Growth of Primary Tumors in Syngeneic Mouse Models

We proposed that the reduced levels of plasma membrane PD‐L1 in 6J1‐treated tumor cells might render them more susceptible to attack by T cells. To address this possibility, we performed an in vitro T cell‐mediated tumor cell killing assay to assess if 6J1 might increase CD8^+^ T cell‐mediated cytotoxicity against 4T1 mouse mammary carcinoma cells in vitro. We showed that when used at concentrations ≤ 1 µm, 6J1 exhibited little effect on the proliferation of CD8+ T cells (Figure S4K, Supporting Information) or 4T1 cells (Figure S4L, Supporting Information). However, 6J1‐treated 4T1 cells were more sensitive to pre‐activated T cell‐mediated cytolysis, when compared with the control cells (Figure 3G, Figure S4M, Supporting Information). These results suggest that 6J1 treatment of tumor cells increases cocultured CD8^+^ T cell‐mediated cytotoxicity.

We also performed a pharmacokinetic (PK) analysis after oral or tail‐vein delivery of 6J1 in rats. The maximum plasma concentration (*C*_max_) of 6J1 after tail‐vein or oral delivery (15 mg kg^−1^) was 10052 ± 1551 ng mL^−1^ (1.8 µm) and 1046 ± 541 ng mL^−1^ (1.78 µm), respectively, and the bioavailability of oral 6J1 was approximately 43.3% (Figure 3H, and Tables S1 and S2, Supporting Information). The PK results indicate that oral 6J1 administration might produce adequate drug exposure.

**Figure 4:**
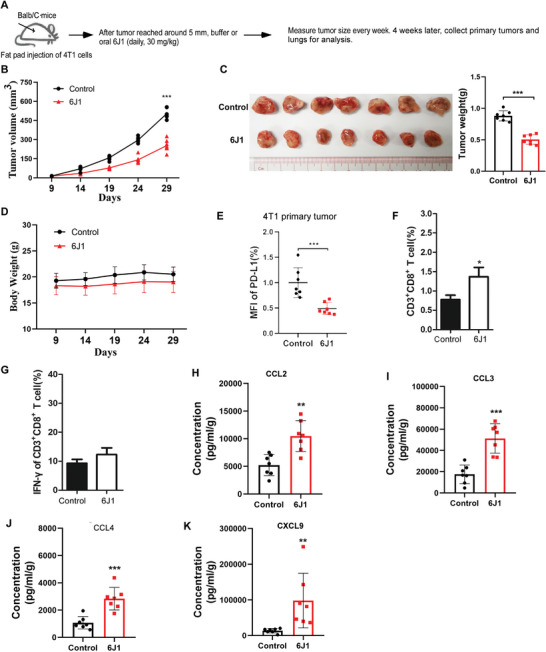
6J1 suppresses the growth of primary tumors in *a* syngeneic mouse model. A–D) 4T1 cells were injected into the fat pads of female Balb/c mice. The mice were randomly divided into two groups (n = 7 per group) and treated with either buffer or 6J1 (30 mg kg^−1^, daily) via oral gavage (A). The tumor volume (B) and body weight (D) of the mice were measured at the indicated time points. At the end time point, representative images of mouse tumors were taken, and the tumor weight was quantified (C). The primary tumors were also isolated at the end of time points. E) Flow cytometry‐based quantification of the levels of plasma membrane PD‐L1 in the primary tumors was performed. F) CD3^+^CD8^+^ T‐cell populations and G) intracellular levels of the cytokine IFN‐*γ* in isolated tumor‐infiltrating lymphocytes were assessed by flow cytometry. H‐K) In addition, CCL2, CCL3, CCL4, and CXCL9 secreted in the microenvironment of tumors were examined by flow cytometry. Data are reported in pg mL^−1^, standardized to the excised tumor weight (in grams); each dot represents one mouse. The graphs represent mean ± s.e.m of three independent experiments, and the asterisks indicate significant differences at *P < 0.05, **P < 0.01, or ***P < 0.001. “ns” indicates data that are not significantly different.

We subsequently examined whether 6J1 could change the TME or the anticancer immunity in an orthotopic breast cancer mouse model by injection of 4T1 cells in the mammary fat pad of syngeneic Balb/c mice. After tumors were palpable (at approximately day 9), the mice were treated with 6J1 (by oral gavage) (30 mg kg^−1^) daily for 4 weeks, after which the mice were euthanized, and the primary tumors were harvested for analysis. We showed that 6J1 significantly decreased the growth of the tumor (**Figure** **4**A–C), but did not change the bodyweight of the mouse (Figure 4D). We then isolated the primary tumors from 4T1‐implanted mice treated with or without 6J1, and found that 6J1 significantly decreased the levels of PD‐L1 at the cell surface of the primary tumor cells (Figure 4E). We also analyzed the CD8+ T cells in these primary tumor tissue. Interestingly, 6J1 increased the percentage of tumor‐infiltrating CD8+ T cells (Figure 4F), but only slightly increased the expression of IFN‐*γ* in the primary tumor tissues, when compared with the control groups (Figure 4G). These results suggest that there might be a high level of CD8+ T cell exhaustion in the microenvironment of 6J1‐treated 4T1 tumors. We further measured the chemokines or cytokines in these primary tumor tissues. We showed that in the TME of 4T1 tumors, 6J1 significantly increased the secretion of some chemokines (e.g., CCL2, CCL3, CCL4, CXCL9, and CXCL10) (Figure 4H–K), but it had little or only subtle effects on others (e.g., CCL5, CCL11, CCL17, CCL22, CXCL1, CXCL5, and CXCL13) (Figure S5A, Supporting Information). In vitro, 6J1 also markedly induced the secretion of various chemokines from 4T1 cells and Raw246.7 cells (Figure S5B, S5C, Supporting Information). These results suggest that the ability of 6J1 to induce the secretion of inflammatory cytokines might turn the immune‐cold microenvironment of 4T1 tumors into immune‐hot, thereby recruiting more cytotoxic T cells to it, but these T cells appear to be exhausted.

We also examined the anticancer effect of 6J1 in a melanoma xenograft mouse model, by the subcutaneous injection of B16‐F10 cells in syngeneic C57BL mice (Figure S6A, Supporting Information). We showed that 6J1 markedly inhibited the growth of B16‐F10 tumors (Figure S6B, S6C, Supporting Information), without affecting the body weight of the animal (Figure S6D, Supporting Information). In addition, it significantly decreased the levels of PD‐L1 at the cell surface of the primary tumors (Figure S6E, Supporting Information). In B16‐F10 tumors, similar to 4T1 tumors, 6J1 also markedly increased the percentage of tumor‐infiltrating CD8+ T cell populations (Figure S6F, Supporting Information), but only subtly increased the expression of IFN‐*γ* (Figure S6G, Supporting Information) when compared with the control groups. Notably, 6J1 did not affect the growth of primary tumors in nude mice implanted with 4T1 or B16‐F10 cells (Figure S6H, S6I, Supporting Information). Nevertheless, these results indicate that 6J1 might inhibit the growth of primary tumors in vivo, which is likely dependent on the intact immune system.

### 2.5 6J1 Induces PD‐L1 Secretion via EVs by Activating Rab27

It is intriguing that following 6J1 treatment of 4T1 or B16‐F10 tumors, there appeared to be CD8+ T cell exhaustion in the TME, manifested by the low expression levels of IFN‐*γ* in these cells (Figure 4G, Figure S6G, Supporting Information). We speculated that in addition to its effects on PD‐L1, 6J1 might also compromise the endosomal trafficking of cell surface MHC‐I in tumor cells. If this was the case, then it would offset the ability of 6J1 to increase T cell‐mediated immunity via the PD‐L1 route. However, 6J1 treatment failed to affect the levels of cell surface MHC‐I in cancer cells (Figure S7A, S7B, Supporting Information).

**Figure 5:**
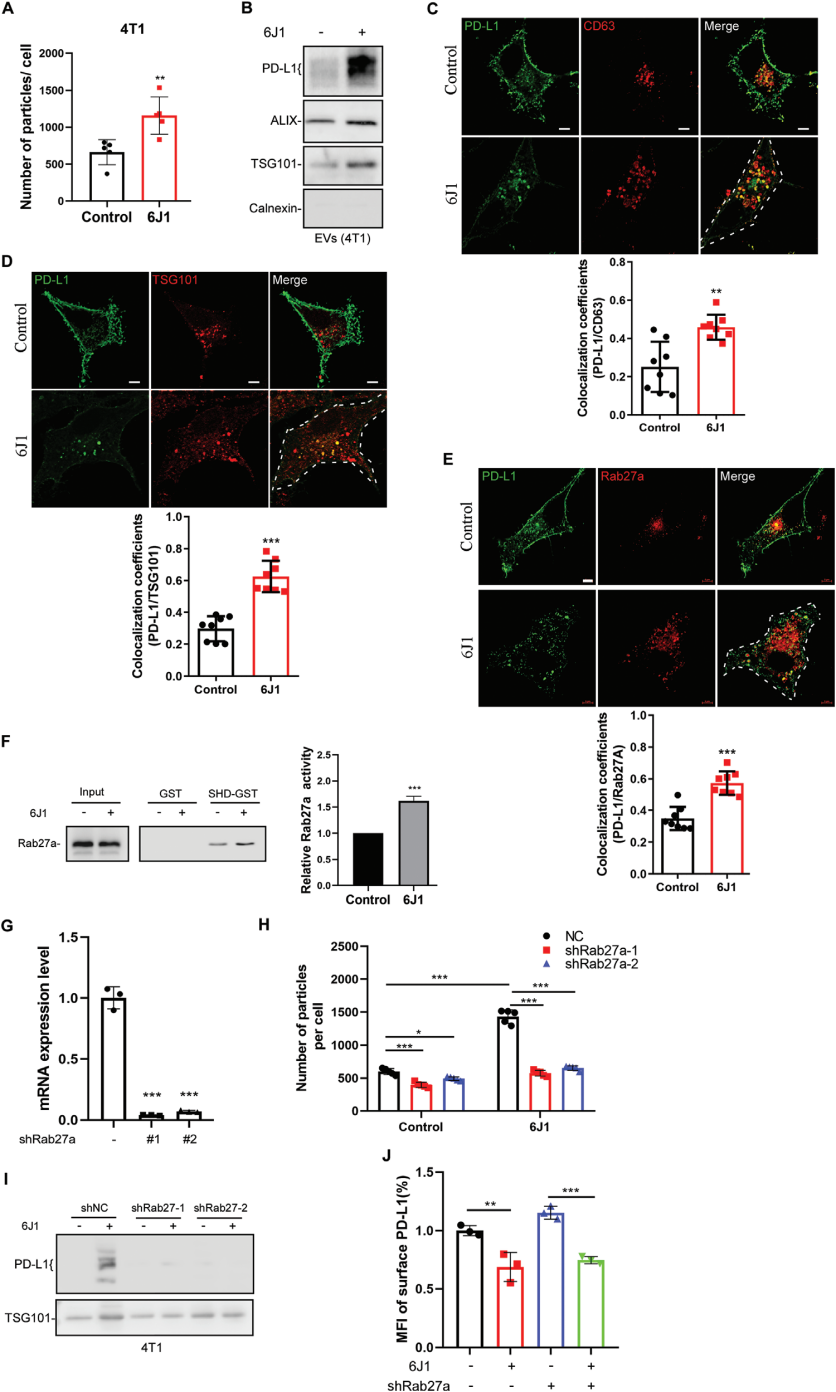
6J1 induces PD‐L1 secretion in EVs via Rab27. A) 4T1 cells were treated with or without 6J1 (1 µm) for 24 h, after which the supernatant was collected, and the concentration of EVs was determined using a Nanosight nanoparticle analyzer. B) 4T1 cells were treated with or without 6J1 (1 µm) for 48 h, after which the EVs were purified from the supernatant and subjected to western blot analysis. C–E) PD‐L1‐GFP‐expressing HeLa cells were transfected with CD63‐mcherry (C), TSG101‐mcherry (D), or Rab27a‐mcherry (E). Cells were then treated with or without 6J1 (1 µm) and fixed after 24 h. MCCs of PD‐L1 with CD63 (C), TSG101 (D), or Rab27 (E) were quantified. The scale bar is 5 µm. F) The activity of Rab27a was examined with a GST–SHD pulldown assay in cells treated with or without 6J1 (1 µm) for 24 h. G) qRT‐PCR analysis of Rab27a expression in 4T1‐shRab27a cells. H,I) 4T1‐shNC or 4T1‐shRab27a cells were treated with or without 6J1 (1 µm). The supernatant was collected, and the concentration of EVs was determined using the Nanosight nanoparticle analyzer (H). The EVs were also purified from the supernatant and subjected to western blot analysis of an EV marker and PD‐L1 (I). J) Flow cytometry‐based quantification of the plasma membrane levels of PD‐L1 in 4T1‐shNC or 4T1‐shRab27a cells treated with or without 6J1 (1 µm). In (A, C–H, J), the graphs represent mean ± s.e.m of three independent experiments, and the asterisks indicate significant differences at *P < 0.05, **P < 0.01, ***P < 0.001. “ns” indicates data that are not significantly different.

Interfering with endolysosomal trafficking has been shown to promote EV secretion.^[^ ^24^ ^]^ Therefore, we reasoned that 6J1 might induce the EV secretion of any PD‐L1 trapped in the endocytic vesicles, and this increased level of PD‐L1 in EVs might interact with PD‐1 in T cells and inhibit their activity. Indeed, we (and others) showed that in various tumor cells, PD‐L1 was secreted in EVs (Figure 2D–F, Figure S2F, S2G, Supporting Information).^[^ ^25^ ^]^ In addition, PD‐L1 in EVs has been reported to suppress the function of CD8 T cells, and hence facilitate tumor growth.^[^ ^25^, ^26^ ^]^ We, thus, quantified the concentration of EVs in the culture medium of control or 6J1‐treated cells with a nanoparticle analyzer. Indeed, 6J1 markedly increased the secretion of EVs in 4T1 and HeLa cells (**Figure** **5**A, Figure S7C, Supporting Information). We then purified the EVs from the supernatant of tumor cells treated with or without 6J1 and analyzed the levels of PD‐L1 by immunoblot analysis. We found that 6J1 markedly increased the levels of PD‐L1, Alix, and TSG101 in EVs (Figure 5B, Figure S7D, Supporting Information). Likewise, 6J1 significantly induced the colocalization of PD‐L1 with the EV markers, CD63, TSG101, and Rab27a (Figure 5C–E). Whereas, 6J1 markedly decreased the colocalization between PD‐L1 and the recycling endosome marker, Rab11 (Figure S7E, Supporting Information). In summary, these results indicate that 6J1 not only induces the accumulation of PD‐L1 at early endosomes, but also increases the EVs secretion of PD‐L1.

We reasoned that the increase in the secretion of PD‐L1‐containing EVs from cells might lead to a decrease in its total protein level in 6J1‐treated cells. Indeed, 6J1 treatment of 4T1 cells markedly decreased the levels of PD‐L1 when compared with the untreated controls (Figure S7F, Supporting Information). However, treatment of 4T1 cells with MG132 (a proteasome inhibitor), Baf A1, or chloroquine (a lysosomal inhibitor), failed to abolish the 6J1‐mediated decrease of PD‐L1 levels in cells (Figure S7G, S7H, Supporting Information). On the other hand, Rab27a knockdown (Figure S7I, Supporting Information) or GW4869 (an exosome secretion inhibitor) (Figure S7J, Supporting Information) rescued PD‐L1 levels in 6J1‐treated cells. These data, therefore, indicate that 6J1 likely reduces the level of PD‐L1 in cells, by increasing EVs secretion rather than via proteasomal or lysosomal degradation.

Since PD‐L1 in EVs might suppress the tumor‐infiltrating T cell cytotoxicity in vivo, we explored ways to block the 6J1‐induced EVs secretion of PD‐L1 without affecting its ability to decrease the levels of cell surface PD‐L1. Since 6J1 significantly induced the colocalization of PD‐L1 and Rab27a (Figure 5E), we examined if it might activate Rab27 to induce EVs secretion. We thus performed a GST‐Synaptotagmin‐like Protein Homology Domain (SHD) pulldown assay as the SHD of the Rab27 effector (Slac2‐b) specifically binds to GTP‐bound Rab27.^[^ ^27^ ^]^ We showed that 6J1 significantly increased the Rab27 GTP activity (Figure 5F). We subsequently conducted shRNA‐mediated knockdown of Rab27 in 4T1 cells (Figure 5G), and showed that 6J1‐induced EVs secretion (Figure 5H) and PD‐L1 levels in EVs (Figure 5I) were abolished in these 4T1‐shRab27a cells. However, Rab27 knockdown did not affect either the 6J1‐induced decrease of PD‐L1 at the cell surface (Figure 5J), or the proliferation of 4T1 cells (Figure S7K, Supporting Information). These results suggest that 6J1‐induced PD‐L1 secretion in EVs is Rab27‐dependent.

### 2.6 The Ability of 6J1 to Promote Anticancer Immune Responses is Enhanced by the Inhibition of EV Secretion

**Figure 6:**
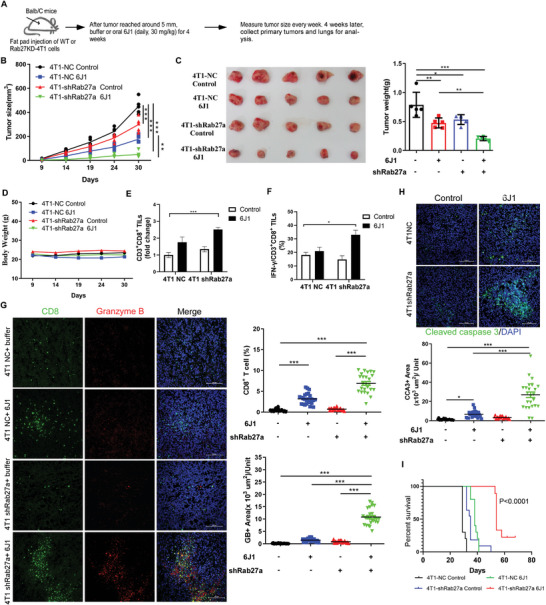
Blocking EVs secretion enhances the anticancer activity of 6J1 in the Balb/c orthotopic mouse model. A–D) 4T1‐shNC or 4T1‐shRab27a cells were injected into the fat pads of female Balb/c mice. The mice were randomly divided (n = 5 per group) and treated with either buffer or biotin‐V1 (30 mg kg^−1^, daily) via oral gavage. The tumor size (B) and the body weight of the mice (D) were measured at the indicated time points. At the final time point, images were collected to show the morphology of the tumors, and the tumor weight was quantified (C). E,F) The CD3+CD8+ T‐cell populations (E) and levels of intracellular IFN‐*γ* (F) in isolated tumor‐infiltrating lymphocytes (TILs) were assessed by flow cytometry. G,H) The expression of CD8 and granzyme B (G), and cleaved caspase 3 (H) in the primary tumors were identified by immunolabeling. I) Line graph showing the percent survival of mice bearing 4T1‐shNC or 4T1‐shRab27a tumors with or without 6J1 treatment. Significance was determined using the log‐rank test. In (G, H), scale bars are 100 µm. In (B–H), the graphs represent mean ± s.e.m of three independent experiments, and the asterisks indicate significant differences at *P < 0.05, **P < 0.01, ***P < 0.001.

To evaluate if the inhibition of EVs secretion by Rab27 knockdown enhances the antitumor efficacy of 6J1, we orthotopically implanted control or Rab27‐knockdown 4T1 cells into syngeneic Balb/c mice. The mice were then treated with 6J1 (30 mg kg^−1^, oral, daily) for 4 weeks (**Figure** **6**A). We showed that in Rab27‐knockdown 4T1 cell‐implanted mice treated with 6J1, tumor growth and burden were both significantly lower when compared with either control 4T1 cell‐implanted mice treated with or without 6J1, or Rab27‐knockdown 4T1 cell‐implanted mice without 6J1 treatment (Figure 6B–D). Moreover, the population of tumor‐infiltrating CD8+ T cells and expression of IFN‐*γ* were both significantly increased in the primary tumor tissues isolated from Rab27‐knockdown 4T1 cell‐implanted mice treated with 6J1, when compared with those in the other groups of mice (Figure 6E,F). We also performed double immunostaining of CD8 and granzyme B in sections prepared from the primary tumors isolated from 4T1 cell‐implanted mice. Consistently, an increased number of infiltrated CD8+ T cells and stronger granzyme B signals were detected in the tumor sections isolated from 6J1‐treated mice implanted with Rab27‐knockdown 4T1 cells, when compared with those from other groups (Figure 6G). Since enhanced anticancer immunity leads to increased apoptosis in tumor cells,^[^ ^28^ ^]^ we assessed the levels of cleaved caspase 3 (CCA3) in tumor sections isolated from mice implanted with 4T1 cells. We found that higher levels of CCA3 indicating enhanced apoptosis were indeed detected in tumor sections from Rab27‐knockdown 4T1 cell‐implanted mice treated with 6J1, when compared with other groups (Figure 6H). Moreover, the combination of Rab27 knockdown and oral 6J1 treatment substantially prolonged the overall survival of mice bearing orthotopic 4T1 tumors, when compared with the other groups (Figure 6I). Together, these results suggest that blocking PD‐L1 secretion in EVs increases the anticancer immune response of 6J1.

We also implanted control or Rab27‐knockdown 4T1 cells into athymic nude mice, followed by 6J1 treatment (30 mg kg^−1^, oral, daily) (Figure S8A, Supporting Information). We showed that 6J1 treatment or Rab27 knockdown had little effect on tumor growth and tumor burden in these immunocompromised mice (Figure S8B–S8D, Supporting Information), which again suggests that the ability of 6J1 to control tumor growth is dependent on an intact immune system.

In addition, we showed that treatment of cells with GW4869, a sphingomyelinase inhibitor that can abolish the secretion of EVs,^[^ ^29^ ^]^ significantly inhibited 6J1‐induced EVs secretion (Figure S8E, Supporting Information), but did not affect the ability of 6J1 to decrease the levels of cell surface PD‐L1 (Figure S8F–S8H, Supporting Information). Therefore, we assessed the combinatorial effects of 6J1 and GW4869 on the growth of primary tumors in an orthotopic cancer mouse model, by injecting 4T1 cells into the fat pads of Balb/C mice (Figure S8I, Supporting Information). We showed that together, GW4869 and 6J1 suppressed tumor growth and decreased tumor burden more effectively than when either GW4869 or 6J1 were applied alone (Figure S8I–S8K, Supporting Information). In summary, these results indicate that the inhibition of EVs secretion in tumor cells significantly augments the ability of 6J1 to promote anticancer immune response in vivo.

### 2.7 A Combination of 6J1 and Anti‐PD‐1 Antibody Promotes Anticancer Immune Responses

**Figure 7:**
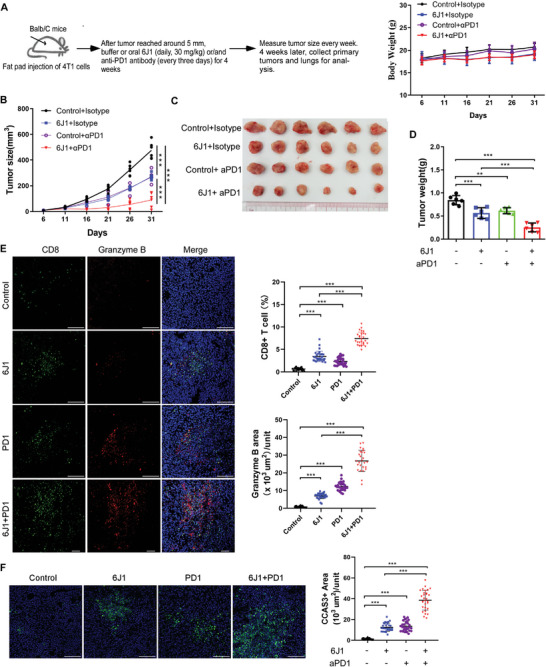
A combination of 6J1 and anti‐PD‐1 antibody promotes anticancer immune responses. 4T1 cells were injected into the fat pads of female Balb/c mice. The mice were randomly divided (n = 6 per group) and treated with either buffer or V1 (30 mg kg^−1^, daily, oral gavage) and/or PD1 antibody (200 µg/per mouse, every 3 days, IV). A,B) The body weight of the mice (A) and tumor volume (B) were measured at the indicated time points. C,D) At the end time point, images were acquired of the mouse tumors following each of the four treatment regimes (C), and the weight of each tumor was determined (D). E,F) In the primary tumors, the expression of CD8 and granzyme B (E), and cleaved Caspase3 (F) was determined by immunolabeling. In (E, F), the scale bars are 100 µm. In (B, D–F), the graphs represent mean ± s.e.m of three independent experiments, and the asterisks indicate significant differences at **P < 0.01 or ***P < 0.001.

Our results suggest that 6J1 induces the secretion of PD‐L1 in EVs into the TME, and this EVs‐containing PD‐L1 might bind to PD‐1 on T cells and in this way suppress their antitumor activity (Figures 4 and 5, and Figures S5–S8, Supporting Information). We hypothesized that a neutralizing anti‐PD‐1 antibody might be able to prevent such PD‐L1/PD‐1 binding, and so a combination of 6J1 and anti‐PD‐1 antibody might exhibit better anticancer activity than either of these treatments alone. To test this, we injected 4T1 cells into the mammary fat pad of syngeneic Balb/C mice, followed by treatment with 6J1 alone, anti‐PD‐1 antibody alone, or a combination of both (**Figure** **7**A). The tumor growth and tumor burden in 4T1 cell‐implanted mice treated with both 6J1 and anti‐PD‐1 antibody were significantly weaker when compared with 4T1 cell‐implanted mice treated with 6J1 or anti‐PD‐1 antibody alone (Figure 7B–D). Moreover, an increased number of infiltrated CD8+ T cells and stronger granzyme B signals were detected in tumor sections isolated from mice treated with both 6J1 and anti‐PD‐1 antibody, when compared to those from mice treated with 6J1 or anti‐PD‐1 antibody (Figure 7E). Likewise, the larger areas of CCA3 signal were detected in tumor sections from 4T1‐implanted mice treated with both 6J1 and anti‐PD‐1 antibody, when compared with either treatment alone (Figure 7F). These results demonstrate that 6J1 promotes the anticancer immunity of anti‐PD‐1 antibody, and vice versa. Therefore, manipulation of PD‐L1 endosomal trafficking by small chemicals provides an effective strategy to promote anticancer immunity, in addition to the existing ICIs.

### 2.8 The In Vivo Toxicity of 6J1 is Low

Last, we investigated the in vivo toxicity of 6J1 in mice. Briefly, 6J1 (30 mg kg^−1^) was given daily via oral route to young adult male and female mice for four weeks. Clinical observations, for example, food consumption, body weight changes, and ophthalmic exams, were performed every three days. The 6J1‐treated mice showed no signs of behavior abnormality. 6J1 had no effects on mouse weight gain and weights of various organs (Figure S9A, S9B, Supporting Information). 6J1‐treated mice showed no apparent changes in liver function test (Figure S9C, Supporting Information). Also, no signs of pathological changes in major organs of 6J1‐treated mice were identified (Figure S9D, Supporting Information). In summary, these data indicate that the in vivo toxicity of 6J1 at a therapeutic dose in mice is low.

We also evaluated the toxicity profile following oral gavage administration of 6J1 at dose levels of 0 (vehicle), 150, 450, and 1000 mg kg^−1^ once daily for up to 7 consecutive days in rats. We found that repeated daily dosing with 6J1 at dose levels ≥450 mg kg^−1^ was not tolerated as it was associated with mortality (1000 mg kg^−1^) and severe clinical findings (450 and 1000 mg kg^−1^) requiring moribund termination (450 and 1000 mg kg^−1^) and interim termination (1000 mg kg^−1^) of the treatment phase. Although repeated daily dosing (7 consecutive days) with 6J1 was considered tolerated up to 300 mg kg^−1^, 6J1 (300 mg kg^−1^)‐related clinical signs were noted primarily towards the end of dosing (soft feces, hunched posture, and reduced feces) and baseline (Day‐1) body weight loss not exceeding 8% by Day 7 (Figure S9E, Supporting Information). Nevertheless, these results indicate that the therapeutic window of 6J1 in experimental animals is high.

## 3 Discussion

**Figure 8:**
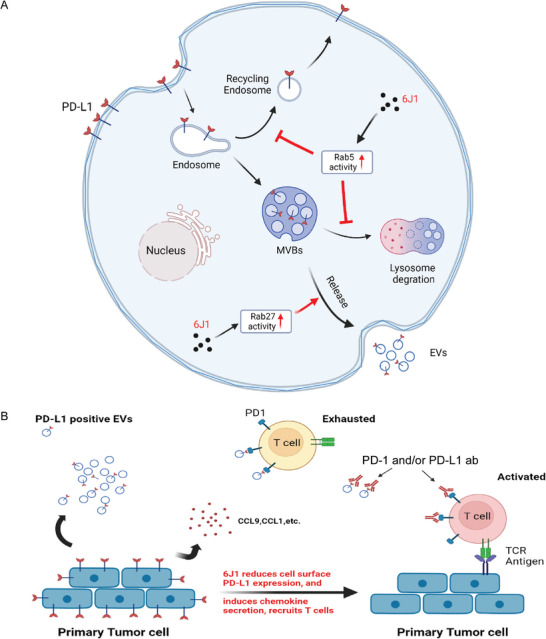
Model of how 6J1 compromises the endosomal trafficking of PD‐L1 to promote anticancer immunity. A) Plasma membrane PD‐L1 is subjected to a constitutive endosomal trafficking process. After internalization, PD‐L1 is trafficked from the early endosomes to either recycling endosomes or MVBs/late endosomes, which are either trafficked to lysosomes or secreted via EVs. The triazine compound, 6J1, blocks the endosomal trafficking of PD‐L1 by activating Rab5 and induces its secretion in EVs by activating Rab27. This ultimately leads to a decrease of PD‐L1 at the plasma membrane. B) 6J1 increases the secretion of some chemokines to facilitate the recruitment of tumor‐infiltrating CD8+ T cells to the TME. A neutralizing anti‐PD‐1 or anti‐PD‐L1 monoclonal antibody could prevent PD‐L1 (both EVs‐containing and plasma membrane PD‐L1) /PD‐1 binding. A combination of 6J1 with a PD‐1 monoclonal antibody significantly promotes the anticancer immune response.

One significant finding of this current study is the characterization of the previously unappreciated role of PD‐L1 endosomal trafficking in tumor cells. We demonstrated that plasma membrane PD‐L1 is subjected to a constitutive endosomal trafficking process. Indeed, once internalized, this protein is trafficked from the early endosomes to either recycling endosomes or MVBs/late endosomes, after which it is either trafficked to lysosomes or secreted via EVs. The endosomal trafficking of PD‐L1 is dependent on Rab5. Another important finding from this study is the application of endosomal inhibitors as an effective strategy for promoting anticancer immunity. We demonstrated that the triazine compound, 6J1, blocked the endosomal trafficking of PD‐L1 by activating Rab5 and induced its secretion in EVs by activating Rab27. This ultimately led to a decrease of PD‐L1 at the plasma membrane (model shown in **Figure** **8**A). 6J1 significantly increased the secretion of some chemokines to facilitate the recruitment of tumor‐infiltrating CD8+ T cells to TME. Moreover, a combination of 6J1 with the inhibition of PD‐L1 secretion in EVs or with a PD‐1 monoclonal antibody significantly promoted the anticancer immune response (model shown in Figure 8B). These important results indicate that compromising the endosomal trafficking of PD‐L1 is an effective approach to improving the efficacy of ICIs.

PD‐L1 has previously been shown to be palmitoylated at the plasma membrane, which prevents its mono‐ubiquitination and subsequent degradation in the lysosome.^[^ ^30^ ^]^ On the other hand, Huntingtin interacting protein 1 related (HIP1R) interacts with PD‐L1 and escorts it to the lysosome for degradation.^[^ ^31^ ^]^ CMTM6 and CMTM4 also interact with PD‐L1, both at the plasma membrane and in the recycling endosomes, and this interaction prevents the lysosomal degradation of PD‐L1 and helps maintains it at the cell surface.^[^ ^10^ ^]^ AMP‐activated protein kinase (AMPK), on the other hand, could phosphorylate PD‐L1 to disrupt the interaction between PD‐L1 and CMTM4, thereby resulting in the degradation of PD‐L1.^[^ ^32^ ^]^ Here, however, we showed that within just 60 min after triggering the internalization of plasma membrane PD‐L1 with its antibody, almost all of the PD‐L1‐antibody complex was internalized and trafficked to the early endosomes, recycling endosomes, MVB/late endosomes, and lysosomes (Figure 1C,H). Rab5 knockdown blocked the trafficking of PD‐L1 to these various endocytic vesicles and did not appear to affect its internalization (Figure 1G–I, Figure S1C, S1D, Supporting Information). In addition, the overexpression of Rab5CA locked PD‐L1 in the early endosomes (Figure 1J). These results indicate that plasma membrane PD‐L1 is subjected to a constitutive endosomal trafficking process, and the early endosomes might act as a sorting hub to regulate the destination of the internalized PD‐L1. Whether the posttranslational modifications of PD‐L1 (e.g., phosphorylation, palmitoylation, mono‐ubiquitination, and glycosylation), or the chaperone proteins (e.g., CMTM4/6 and HIP1R), are sorting signals in the early endosomes for its various destinations, still remain to be determined.

It has previously been shown that the activation of Rab5 is required for early endosome maturation, whereas its inactivation is necessary for the early endosome to late endosome transition.^[^ ^33^ ^]^ Therefore, the expression of a dominant‐negative mutant of Rab5 (Rab5^S34N^) blocks early endosome maturation, whereas the expression of Rab5CA (Rab5^Q79L^) blocks the early endosome to late endosome transition resulting in enlarged early endosomes.^[^ ^34^ ^]^ Here, we found that 6J1 activated Rab5 and thus blocked the early endosome to late endosome transition, which resulted in an accumulation of early endosomes (Figure 3A–C, and Figure S4A–S4D, Supporting Information). In this way, 6J1 induced the accumulation of internalized PD‐L1 in the early endosomes (Figure 3C, Figure S4G, Supporting Information) and decreased its levels in the plasma membrane (Figure 3D–F, Figure S4H, S4I, Supporting Information). This is likely the reason why 6J1‐treated tumor cells were more susceptible to cytotoxic T cells in vitro (Figure 3G, Figure S4M, Supporting Information). Moreover, 6J1 inhibited the growth of primary tumors in syngeneic mice (Figure 4B,C, Figure S6B, S6C, Supporting Information) but not in immunodeficient mice (Figures S6H, S6I, S8B, and S8C, Supporting Information), suggesting that an intact immune system is required for the antitumor activity of 6J1.

We showed that 6J1 markedly increased the number of tumor‐infiltrated cytotoxic T cells in 4T1 mouse mammary carcinoma‐implanted syngeneic mice (Figures 4F, 6E, and 7E and Figure S6F, Supporting Information). The increased levels of various proinflammatory chemokines (e.g., CCL2, CCL3, CCL4, and CXCL9) found in the TME following 6J1 treatment (Figure 4H–K) might facilitate such infiltration. Likewise, it has previously been shown that tumor‐derived CCL2 trigger effector T cell chemotaxis both in vitro and in vivo,^[^ ^35^ ^]^ and CCL3 and CCL4 can also induce the recruitment of effector T cells.^[^ ^36^ ^]^ We suspect that 6J1 might activate Rabs (e.g., Rab5 or Rab27), to induce the secretion of chemokines or cytokines. Nevertheless, the mechanism underlying 6J1‐induced chemokine production remains to be determined. Notably, it has been speculated that tumors that are not infiltrated by cytotoxic T cells are less likely to respond to ICIs, whereas those that contain pre‐existing T cells are likely to be more responsive to ICIs.^[^ ^37^ ^]^ Indeed, we showed that 6J1‐treated tumors are more sensitive to the PD‐1 blocking antibody, and their combined use significantly promoted anticancer immune responses (Figure 7 and model shown in Figure 8B).

Unexpectedly, the levels of IFN‐*γ* and granzyme B in most of the 6J1‐treated tumors were low (Figures 4G, 6F,G, and 7E, Figure S6G, Supporting Information), which suggests that the majority of T cells were actually exhausted. It has been reported that tumor cell‐derived exosomal PD‐L1 can inhibit tumor‐specific T‐cell activity.^[^ ^24^, ^25^, ^38^ ^]^ In addition, we showed that 6J1 activated Rab27 to induce PD‐L1 secretion in EVs from tumor cells (Figure 5F–J). We suggest, therefore, that in 6J1‐treated tumors, it is likely to be the 6J1 stimulation of PD‐L1 secretion in EVs that is responsible for the T‐cell exhaustion observed. Blocking PD‐L1 secretion in EVs by Rab27 knockdown, significantly revoked the T‐cell activity in the TME and augmented the anticancer activity of 6J1 (Figure 6B–H). Therefore, understanding the mechanisms underlying how 6J1 activates Rab5 or Rab27 could help the identification of a 6J1 analog that specifically activates Rab5, but not Rab27. This analog might effectively promote anticancer immunity alone. Along this line, we have recently identified CapZ as one of the binding proteins of V1 (a 6J1 analog), and demonstrated that V1 targets CapZ to regulate endosomal trafficking.^[^ ^12c,d^ ^]^ It is of interest to determine whether and how these triazine compounds recruit CapZ to early endosomes or MVB to modulate Rab5 or Rab27, respectively, thus regulating endosomal and exosomal trafficking.

Nevertheless, it is clear that the inhibition of PD‐L1 endocytotic trafficking by small chemicals like 6J1 reduces the level of PD‐L1 at the tumor cell surface. More importantly, together our data indicate the potential advantages for developing novel PD‐L1 inhibitors, which when used in combination with existing antibody drugs, might provide new and more effective anticancer therapies.

## 4 Experimental Section

### Cells and Reagents

HeLa cells, B16‐F10, MDA‐MB‐231, Raw246.7, and HEK 293T cells were obtained from ATCC. 4T1 cells were kindly provided by Dr. Minh LE (National University of Singapore). All the cells were maintained in DMEM (Invitrogen, 12800‐017) containing 10% fetal bovine serum (Invitrogen, 10270‐106) and 100 units mL^−1^ of penicillin/streptomycin (Invitrogen, 15140‐122) at 5% CO_2_ and 37 °C. The following antibodies were purchased from Cell Signaling Technology (catalog numbers provided after each name): Anti‐Rab5 (3547), anti‐EEA1 (3288), anti‐LAMP1 (9091), anti‐Rab27a (69 295), anti‐Rab11 (5589), anti‐PD‐L1 (13684), anti‐granzymeB (17215), anti‐ATG5 (2630), and anti‐ATG4B (5299). Anti‐TSG101 (14497‐1‐AP), anti‐CD63 (25682‐1‐AP), anti‐Alix (12422‐1‐AP), anti‐Calnexin (10427‐2‐AP), anti‐GAPDH (60004‐1‐Ig), and anti‐Beta Actin (66009‐1‐Ig) antibodies were purchased from Proteintech. Anti‐PD‐L1 (MIH1, 16‐5983‐38), anti‐PD‐L1 (MIH5, 16‐5982‐38), anti‐CD3 (145‐2C11), and anti‐CD8 (56.6.7) antibodies, DQ‐green BSA (D12050), and Transferrin‐488 (T13342) were purchased from Thermo Fish Scientific. InVivoPlus Anti‐mouse PD1 (29F.1A12) antibody and InVivoPlus rat IgG2a isotype control (2A3) were purchased from Bio X Cell. GW4869 and Bafilomycin A1 were purchased from Selleck. Pipstop2 and Fillipin III were purchased from Cayman.

### Immunofluorescence Staining

When immunolabeling cells for live‐cell imaging, cells were washed with phosphate‐buffered saline (PBS) and incubated with primary antibody on ice for 1 h. They were then washed three times with ice‐cold PBS (containing 0.5% bovine serum albumin; BSA) and incubated with the appropriate fluorescence‐conjugated secondary antibody on ice for 1 h. When immunolabeling fixed cells, cells were fixed with 4% Paraformaldehyde, and then with PBS containing 0.1% Triton X‐100 for 15 min. The permeabilized cells were washed with PBS, blocked with PBS containing 5% BSA for 1 h at room temperature, and then incubated with primary antibody at 4 °C overnight. The cells were washed again and incubated with the appropriate fluorescence‐conjugated secondary antibody for 1 h at room temperature. After another round of washing, the cells were mounted with ProLong Diamond Antifade mountant (Thermo Fisher, P36970). Images were acquired with either a Zeiss LSM 880 confocal microscope or a Nikon A1HD25 High‐Speed and Large‐Field of View Confocal Microscope, after which they were analyzed with the ZEISS ZEN microscopy software or the Nikon NIS Elements AR software, respectively.

### Image Colocalization Analysis

Based on the workflow described by Dunn et al.,^[^ ^39^ ^]^ Manders colocalization coefficient (MCC) or Pearson's correlation coefficient (PCC) was applied to quantify the colocalization of two probes. When analyzing the colocalization between PD‐L1 and different cellular vesicle markers in the PD‐L1‐GFP overexpression cells, such as in Figures 1A and 2A, PCC analysis was used to measure the similarity of shape between two patterns. In this method, the value of 1 indicates that the patterns were perfectly similar; every pixel that contains channel 1 (e.g., PD‐L1) also contains channel 2 (e.g., Rab5). For the endosomal trafficking process of plasma membrane PD‐L1 by live PD‐L1 staining, such as in Figures 1C,H, 2B,C, and 2G, MCC analysis was applied to quantify the fraction of internalized PD‐L1 that colocalizes with different intracellular vesicle markers. MCC quantifies the overlap of two objects located on cellular organelles. In this analysis, the values range from 0 to 1.

### Total Internal Reflection Fluorescence (TIRF) Microscope

For total internal reflection fluorescence microscopy (TIRF‐M), PD‐L1‐GFP‐expressing HeLa cells were seeded on a glass‐bottomed dish. 24 h later, cells were imaged through a 100 × 1.49‐NA TIRF objective on a Nikon STORM (Nikon, Japan) inverted microscope equipped with an iXon EMCCD camera (Andor) and 488 nm Sapphire LP laser (Coherent). Live cells were imaged in a 500 ms interval for 2 min. Cells were maintained at 37  °C and 5% CO_2_ using a stage‐top incubator. Image quantification was conducted using NIS Elements software (Nikon). The fluorescent intensity of ROIs (PD‐L1 puncta) in the cell was quantified at different time points, and this was applied to the single image for each time point.

### Immunohistochemistry of Tumors

Mouse tissue samples isolated from mice were fixed with 10% neutral buffered formalin overnight and dehydrated. They were then either embedded in paraffin (for the preparation of 5 µm serial sections), or else they were immediately frozen in OCT compound (for the preparation of 10 µm serial sections). The sections were then washed with PBS, and blocked with PBS containing 5% BSA for 1 h at room temperature, after which they were incubated with primary antibody at 4 °C overnight. The sections were washed again and incubated with the appropriate fluorescence‐conjugated secondary antibody for 1 h at room temperature. For each tissue sample, 4–5 sections that were acquired in different positions were used for staining and quantification.

### Flow Cytometry

For surface marker analysis, live cells were suspended in PBS containing 1% BSA, and stained with the appropriate antibody at 4 °C for 30 min. For intracellular cytokine staining, cells were fixed and permeabilized, followed by labeling with the indicated antibodies after the cell surface markers staining. The concentration of antibodies used, was according to the product protocol. Data were acquired with a Beckman Coulter CytoFLEX Flow Cytometer and analyzed with the FlowJo 10 software.

### Gene Knockdown or Knockout

shRNA sequences were designed using the Sigma online tool. sgRNA sequences were designed using the Benchling tool (https://www.benchling.com/). shRNAs were cloned into the pLKO.1 lentiviral vector and sgRNAs were cloned into the lentiCRISPR‐V2‐puro vector. Knockout or knockdown cell lines were then generated by infection with lentiCRISPRv2 containing sgRNA (sgRab5: GTGTGATATCATACACAACTA; sgATG4B: AGCAAACCGGA GAGTGTCGT; sgLC3: CGGAGAAGACCTTCAAGCAG; sgRab27: TTCACT TAACTGATCCGTAG for human) or shRNA (shRab27: CCGGGCTTCTGTTCGACCTGACAAACTCGAGTTTGTCAGGTCGAACAGAAGCTTTTT) followed by single‐cell cloning and puromycin selection. The knockout or knockdown efficiency was validated by immunoblot analysis.

### Extracellular Vesicle Purification

Cells were cultured on 15 cm dishes to ≈80% confluency. The cells were then rinsed twice with PBS and incubated with EV‐depleted complete medium containing DMSO (vehicle control) or 6J1 (1 µm) for 48 h. The conditioned medium was then collected, and subjected to sequential centrifugation steps (i.e., 1000 x g for 10 min, 2000 x g for 15 min, and then 10000 x g for 15 min) to remove any dead cells and cell debris. The supernatant was ultracentrifuged at 110000 × g for 90 min. After ultracentrifugation, the resulting supernatant was discarded, and the pellet was resuspended in 10 mL cold PBS and ultracentrifuged again at 110000 × g for 90 min. After this second ultracentrifugation step, the pellet was collected for further analysis.

### Extracellular Vesicle Quantification

The EV number was quantified using a NanoSight Tracking Analysis NS300 system (Malvern, UK). All samples were diluted in PBS to the final per‐frame value (50–200 particles/frame). A total of 0.5 mL of supernatant containing EVs was injected into the sample chamber. Date collecting setting was set according to the manufacturer's software manual. Camera levels were adjusted until all particles were distinctly visible (camera level of 8–12), Autofocus was adjusted so that indistinct particles were avoided. Total of 5 video repetitions of each sample with 60 s capture duration was captured at room temperature. After capture, the in‐build NTA software (NTA 3.2 Dev Build 3.2.16) was used for data processing with a detection threshold of 5 and calculation of the concentration (particles mL^−1^) and particle diameter (nm) for each repetition. The concentration of the EV samples was corrected by the dilution factor.

### Rab27a‐GTP Activity

The Rab27a‐GTP activity was determined by using the GST‐Synaptotagmin‐like Protein Homology Domain (SHD) of the Slac2‐b (hereafter called GST‐SHD) pulldown assay. In brief, cells were lysed in lysis buffer (50 mm HEPES‐KOH (pH 7.2), 80 mm KCl, 4 mm MgCl_2_, 0.2 mm CaCl_2_, 2 mm EGTA, 1 mm dithiothreitol, 2 µm leupeptin, 2.5 µg mL^−1^ trypsin inhibitor, 0.1 mm PMSF, and 2 µg mL^−1^ aprotinin), containing 0.5% Triton X‐100 at 4 °C for 5 min followed by centrifugation at 10 000 x g for 15 min. Then, cell lysates were incubated with an equal amount of glutathione‐Sepharose beads (GE Healthcare Biosciences) coated with 20 µg GST‐SHD fusion protein at 4 °C for 30 min. The beads were then washed four times with lysis buffer (without Triton X‐100) and boiled in SDS‐sample buffer at 95 °C for 5 min to elute GTP‐Rab27a. The GTP‐Rab27a proteins that bound to the beads were analyzed by immunoblotting with the anti‐Rab27a antibody.

### T‐Cell‐Mediated Tumor Cell‐Killing Assay

The spleens of mice were harvested and mechanically dissociated into fragments. Each fragment was ground up using a syringe plunger on a 70‐µm pore‐size cell strainer to prepare single‐cell suspensions. CD8 ^+^ T cells were sorted with anti‐mouse CD8a antibody (Biolegend). Enriched CD8^+^ T cells were then cultured in activation medium, consisting of 50 U interleukin‐2 (rIL‐2) and mouse T cell activator CD3/CD28 (Life Technology, 11452D), for activation and expansion. To analyze the ability of T‐cells to kill tumor cells, 4T1 cells were cocultured with activated CD8^+^ T cells in the presence of DMSO (control) or 6J1. After 60 h, the wells containing the 4T1 cells were washed with PBS twice to remove the T cells, and any surviving tumor cells were quantified with the CCK8 assay.

### Multiplex Bead Arrays for Chemokine Detection

The chemokines in tissue or cell supernatant were determined by a proinflammatory chemokine panel (13X) (Biolegend, 740 451) according to manufacturer protocol. Briefly, tumor tissues were homogenized and centrifuged at 590 g for 5 min at 4 °C to remove residual tissue. Then, samples were incubated with pre‐coated mixed beads for 2 h, and in this way, each analyte could bind to its specific capture beads. After washing, the samples were incubated with a biotinylated detection antibody cocktail for 1 h, followed by incubating with streptavidin‐phycoerythrin for 0.5 h. Data were acquired by Beckman Coulter CytoFLEX Flow Cytometer analyzer, and the intensity was analyzed with LEGENDplex data analysis software. The chemokines, which were below the lowest detection level of kit, were assumed to be undetectable.

### Animal Experiments

BALB/c athymic nude mice and BALB/c female mice were purchased from the Jackson Laboratory (USA) and maintained in pathogen‐free conditions with a 12 h light/dark cycle. All animal studies were performed according to the guidelines approved by the Animal Ethics Committee of the City University of Hong Kong.

4T1 cells (1 × 10^5^) were injected into the right fourth mammary fat pad of 8‐week‐old female BALB/c mice or BALB/c athymic nude mice. When the tumors reached ≈5 mm in length, the mice were randomly divided into different groups, after which they received either the vehicle control (i.e., PEG400/ethanol/tween80 at 1:1:1) or 6J1 orally every day. The primary tumor size (measured with calipers) and total body weight of each mouse were determined every 5 days. At the endpoint, the mice were euthanized, and the tumors were harvested for further analysis.

### Flow Cytometry Analysis of Immune Populations in Tumor Tissues

The tumors were harvested and mechanically dissociated into fragments. Each fragment was ground up using a syringe plunger on a 70‐µm pore‐size cell strainer to prepare single‐cell suspensions, and the red blood cells were lysed with ACK lysis buffer (NH_4_Cl 150 mm, KHCO_3_ 10 mm, Na_2_EDTA 0.1 mm). The remaining cells were counted and stained using the indicated antibodies. Stained samples were analyzed using a BD FACSCanto II (BD Biosciences) cytometer.

### Statistical Analysis

The data were presented as mean ± s.e.m. and statistically significant differences were determined with either the unpaired Student's t‐test or the ANOVA test. The asterisks indicate data that were significantly different at *P* < 0.05 (*); *P* < 0.01 (**), or *P* < 0.001 (***), whereas *P* > 0.05 was considered to be not significantly different.

## Conflict of Interest

This work has applied patent (A METHOD OF PROMOTING ANTITUMOR OR ANTICANCER IMMUNITY; Application number: 17699693). Z.Y., W.P., and J.Y. are the inventors of the patent.

## Supporting information

Supporting Information

Supporting Video 1

## Data Availability

The data that support the findings of this study are available from the corresponding author upon reasonable request.
