# Supplementary material for: Manipulation of PD‐L1 Endosomal Trafficking Promotes Anticancer Immunity
Source: Adv Sci (Weinh). 2022 Dec 25;10(6):2206411. doi: 10.1002/advs.202206411 (PMC9951344; doi:10.1002/advs.202206411)
Supplement: Supplementary file 1 — Supporting Information [file ADVS-10-2206411-s002.pdf]

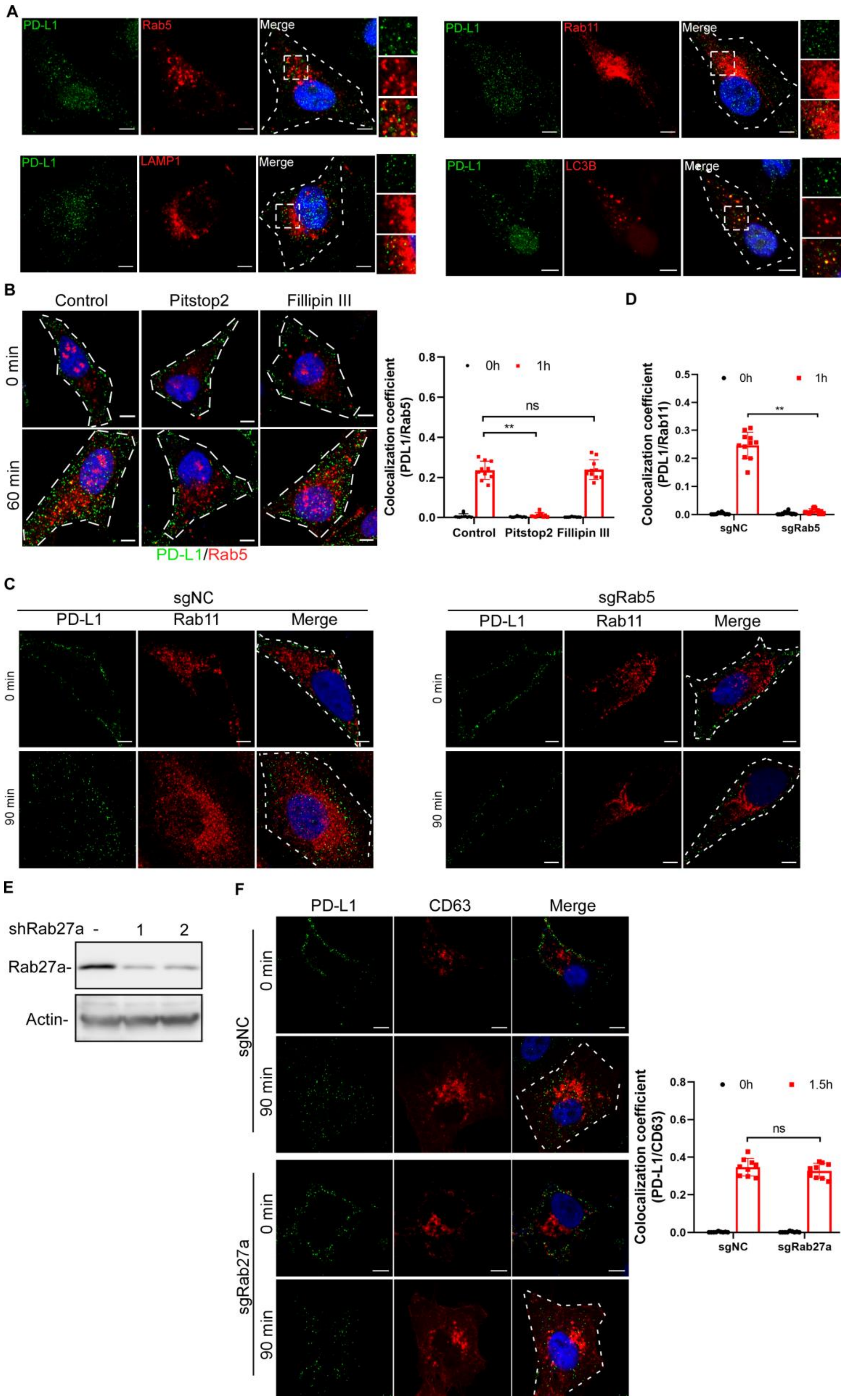
A
PD-L1
Rab5
Merge
Rab11
LAMP1
LC3B
B
Control
Pitstop2
Fillipin III
0 min
60 min
PD-L1/Rab5
Colocalization coefficient (PDL1/Rab5)
0h
1h
ns
**
D
Colocalization coefficient (PDL1/Rab11)
sgNC
sgRab5
C
PD-L1
Rab11
Merge
90 min
E
shRab27a
-
1
2
Rab27a-
Actin-
F
CD63
sgRab27a
Colocalization coefficient (PD-L1/CD63)
1.5h

Figure S1. Plasma membrane PD-L1 undergoes endosomal trafficking. (A) HeLa cells were fixed and co-labeled with antibodies against PD-L1 (green), and either Rab5, Rab11, LAMP1 or LC3B (red). **(B)** HeLa cells were pretreated with Fillilin III (5 mg/mL) or Pistop2 (30 μM) for 3 h, and then incubated on ice for 60 min with the anti-PD-L1 antibody. The cells were then either fixed immediately or else incubated with medium at 37℃ for 60 min prior to fixation. All the fixed cells were labeled with the anti-Rab5 antibody. The colocalization coefficients (MCC) of PD-L1 with Rab5 were quantified. **(C)** Rab5-knockout HeLa cells were incubated on ice for 60 min with the anti-PD-L1 antibody (green). The cells were then either fixed immediately or else incubated with medium at 37℃ for 90 min prior to fixation. All the fixed cells were labeled with an anti-Rab11 antibody (red). **(D)** Quantification of the colocalization (MCC) of PD-L1 and Rab11 from the images in panel **(C)**. **(E)** Western blot analysis of the knockdown efficiency of Rab27a in HeLa cells. **(F)** Rab27a-knockdown HeLa cells were transfected with CD63-mcherry and then incubated on ice for 60 min with the anti-PD-L1 antibody (green). The cells were either fixed immediately or else incubated with medium at 37℃ for 90 min prior to fixation. The colocalization coefficients (MCC) of PD-L1 with CD63 were quantified. In **(A-C, F)**, the scale bars are 5 μm. In **(B, D-F)**, the graphs represent mean ± s.e.m of three independent experiments, and the asterisks indicate significant differences at ** $P < 0.01$. ‘ns’ indicates data that are not significantly different.

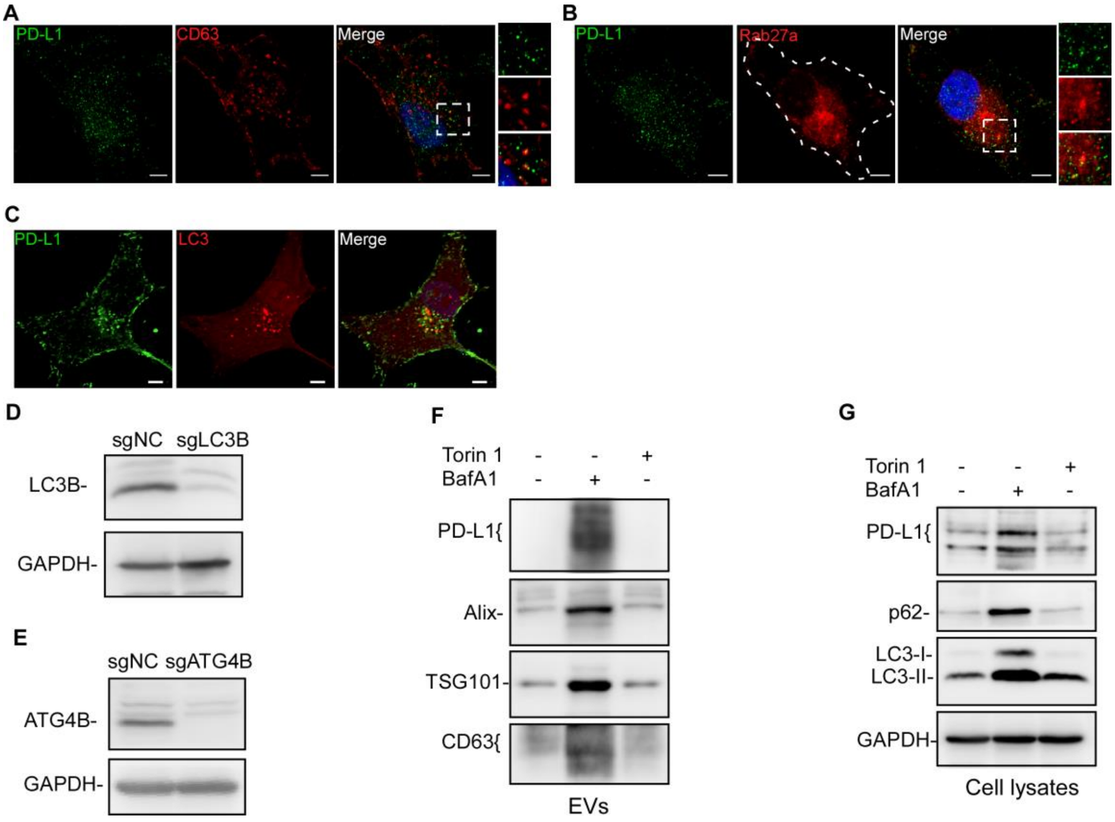

Figure S2. PD-L1 is secreted via EVs. (A) HeLa cells were transfected with

CD63-mcherry (red), after which they were fixed and immunolabeled with anti-PD-L1 antibody (green). **(B)** HeLa cells were fixed and dual-immunolabeled with antibodies against PD-L1 (green) and Rab27a (red). In **(A, B),** the regions bounded by the white dashed squares are shown at higher magnification to the right of the main figure panels, and the scale bars are 5 μm. **(C)** PD-L1-GFP expressing HeLa cells were transfected with LC3B-RFP, after which they were fixed and imaged. The scale bars are 5 μm. **(D)** Western blot analysis of the knockdown efficiency of LC3B in HeLa cells. **(E)** Western blot analysis of the knockdown efficiency of ATG4B in HeLa cells. **(F)** EVs were purified from the supernatant of HeLa cells that had been treated with Bafilomycin A1 (BafA1) (10 nM) or Torin1 (100 nM) for 48 h, and they were subjected to western blot analysis. **(G)** HeLa cells were treated with BafA1 (10 nM) or Torin1 (100 nM) for 24 h, and then cell lysates were collected for western blot analysis.

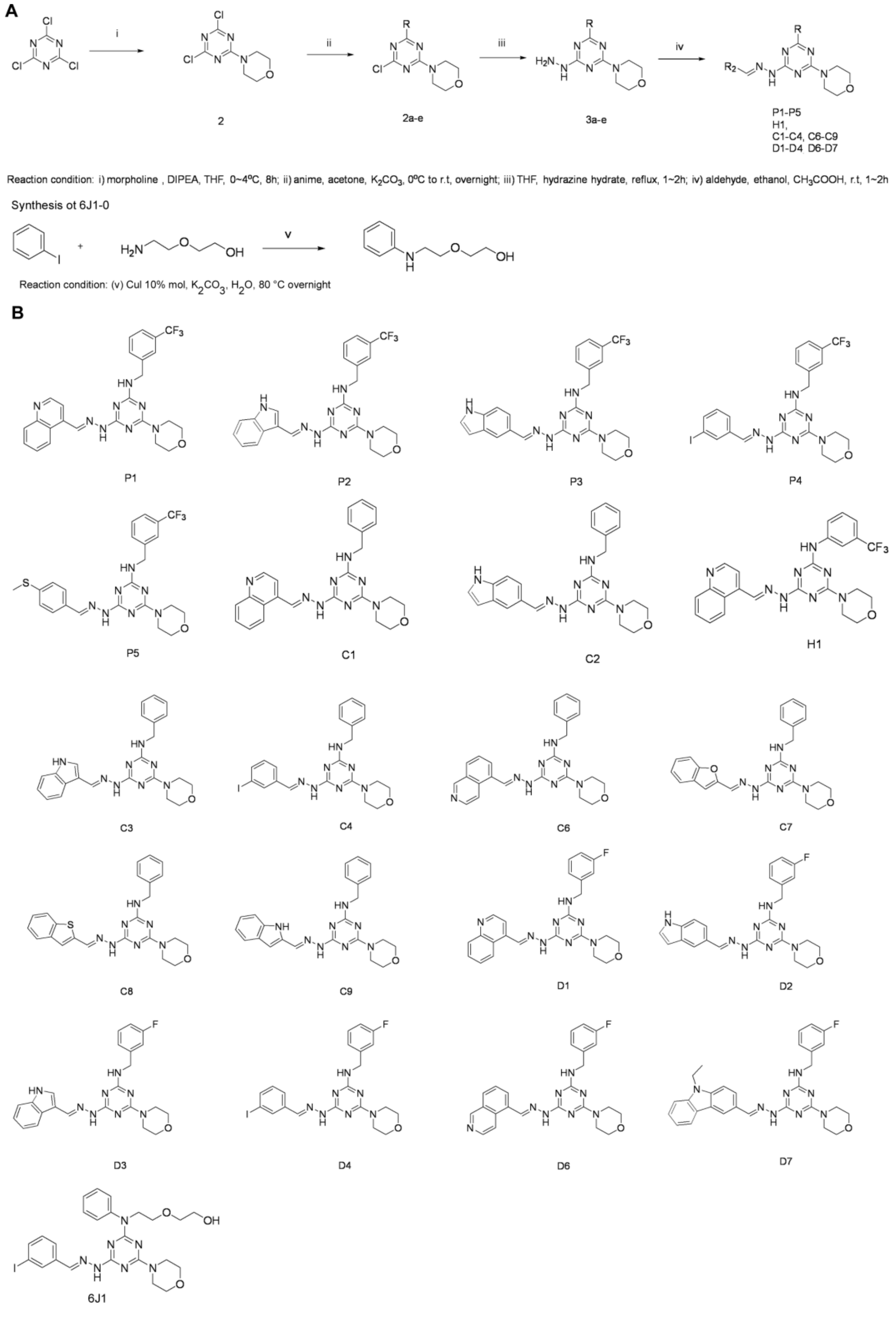

Figure S3. Synthesis of the 6-morpholine-1,3,5-triazine derivatives. (A) Synthetic strategy and route of the 6-morpholine-1,3,5-triazine derivatives. **(B)** Compound structures of the 21 6-morpholine-1,3,5-triazine analogs synthesized.

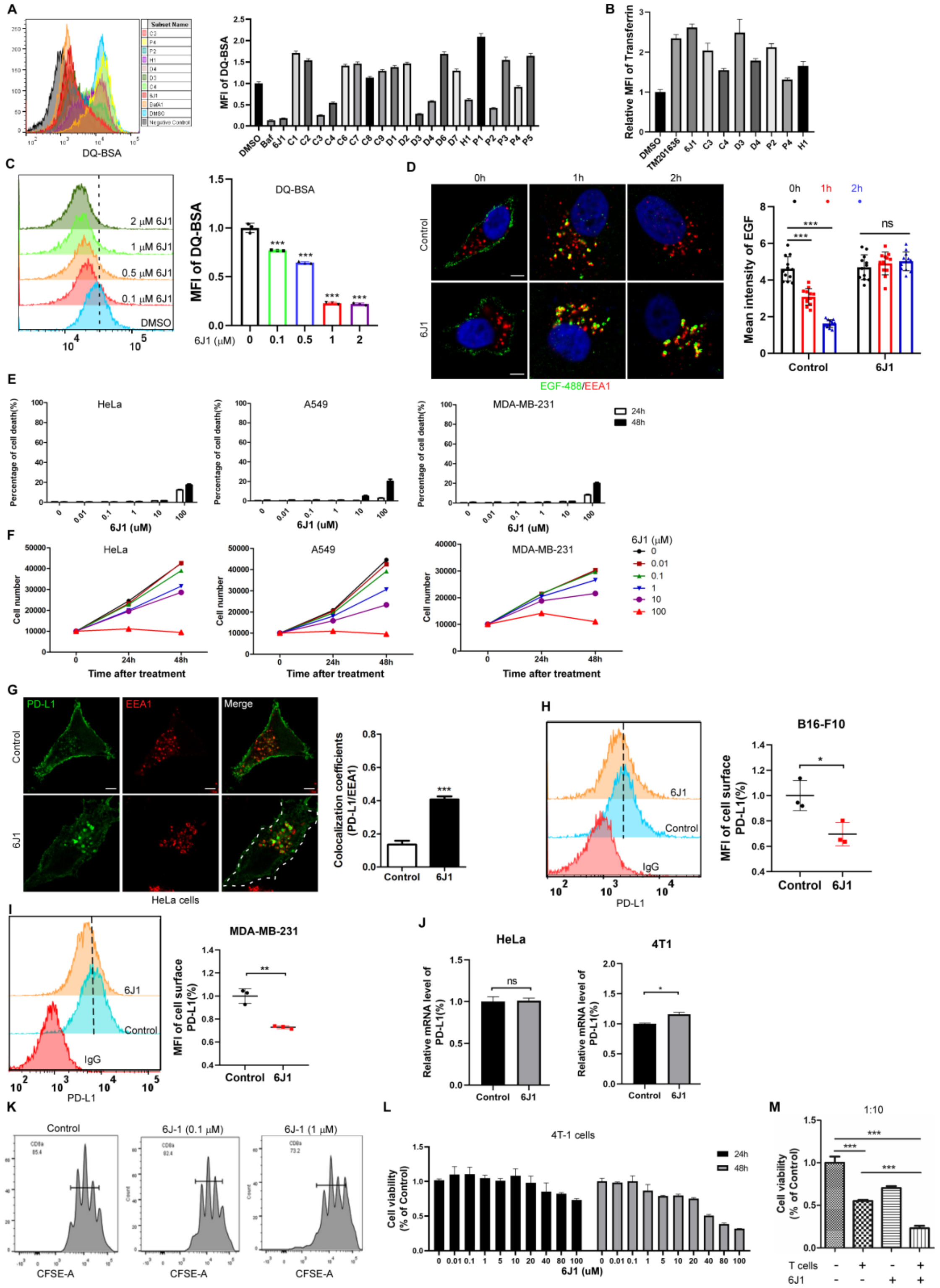

Figure S4. 6J1 is a potent inhibitor of endocytosis and reduces the level of PD-L1 at the cell surface. (A) HeLa cells were plated in 6-well plates and treated with the indicated compound for 3 h. The medium was then replaced with Trafficking media containing 10 μg/ml DQ-green BSA with or without compound (1 μM), and incubated

at 37°C in a 5% CO_2 humidified cell culture chamber for another 6 h, followed by flow cytometry analysis. **(B)** HeLa cells were plated in 6-well plates and treated with the indicated compound for 3 h, after which they were incubated with Transferrin-488 (25 μg/ml) on ice for 1 h, and then in complete medium at 37°C in a CO_2 incubator for 3 h. The cells were collected for flow cytometry analysis, and the mean fluorescence intensity of transferrin was quantified. **(C)** HeLa cells were treated with 6J1 at the indicated concentrations, after which they were subjected to the DQ-green BSA trafficking assay. **(D)** HeLa cells were treated with DMSO or 6J1 (1 μM) for 3 h, after which they were incubated with EGF-488 (2 μg/ml) on ice for 1 h. Some cells were fixed immediately, whereas others were incubated in complete medium at 37°C in a CO_2 incubator for 1 h or 2 h prior to fixation. **(E, F)** Cells were grown in 96-well plates in triplicate and treated with or without 6J1 at the concentrations indicated for 24 h or 48 h. They were then stained with propidium iodide (PI) (**E**) or Hochest 33342 (**F**) to measure the cell death ratio or cell number, respectively. **(G)** PD-L1-GFP (green) expressing HeLa cells were treated with or without 6J1 (1 μM) for 24 h, after which they were fixed and immunolabeled with an anti-EEA1 antibody (red). The colocalization coefficients (MCC) of PD-L1 with EEA1 were quantified. The scale bars in **(D, G)** are 5 μm. **(H-I)** Flow cytometry-based quantification of PD-L1 plasma membrane levels in B16-F10 cells **(H)** or MDA-MB-231 cells **(I)** treated with or without 6J1 (1 μM) for 24 h. The mean fluorescence intensity of PD-L1 was quantified. **(J)** HeLa or 4T1 cells were treated with or without 6J1 (1 μM) for 24 h, after which the levels of PD-L1 mRNA were measured by RT-PCR. **(K)** CD8+ T cells were isolated from mouse spleen, labeled with carboxyfluorescein succinimidyl ester (CFSE; Invitrogen, Germany), and cocultured with rIL-2 (100 U/mL). They were then incubated with 0.1 μM or 1 μM 6J1 for three days, after which the cells were harvested and analyzed by flow cytometry. **(L)** 4T1 cells were plated in 96-well plates in triplicate and treated with or without 6J1 at the concentrations indicated for 24 h or 48 h. The cell viability was then quantified via the MTT assay. **(M)** 4T1 cells were treated with or without 6J1 (1 μM), after which the T-cell-mediated tumor cell-killing assay was determined by CCK8 assay. The graphs represent the mean ± s.e.m of three independent experiments, and the asterisks indicate significant differences at $^*P < 0.05$, $^{**} P < 0.01$ or $^{***}P < 0.001$. 'ns' indicates that data were not significantly different.

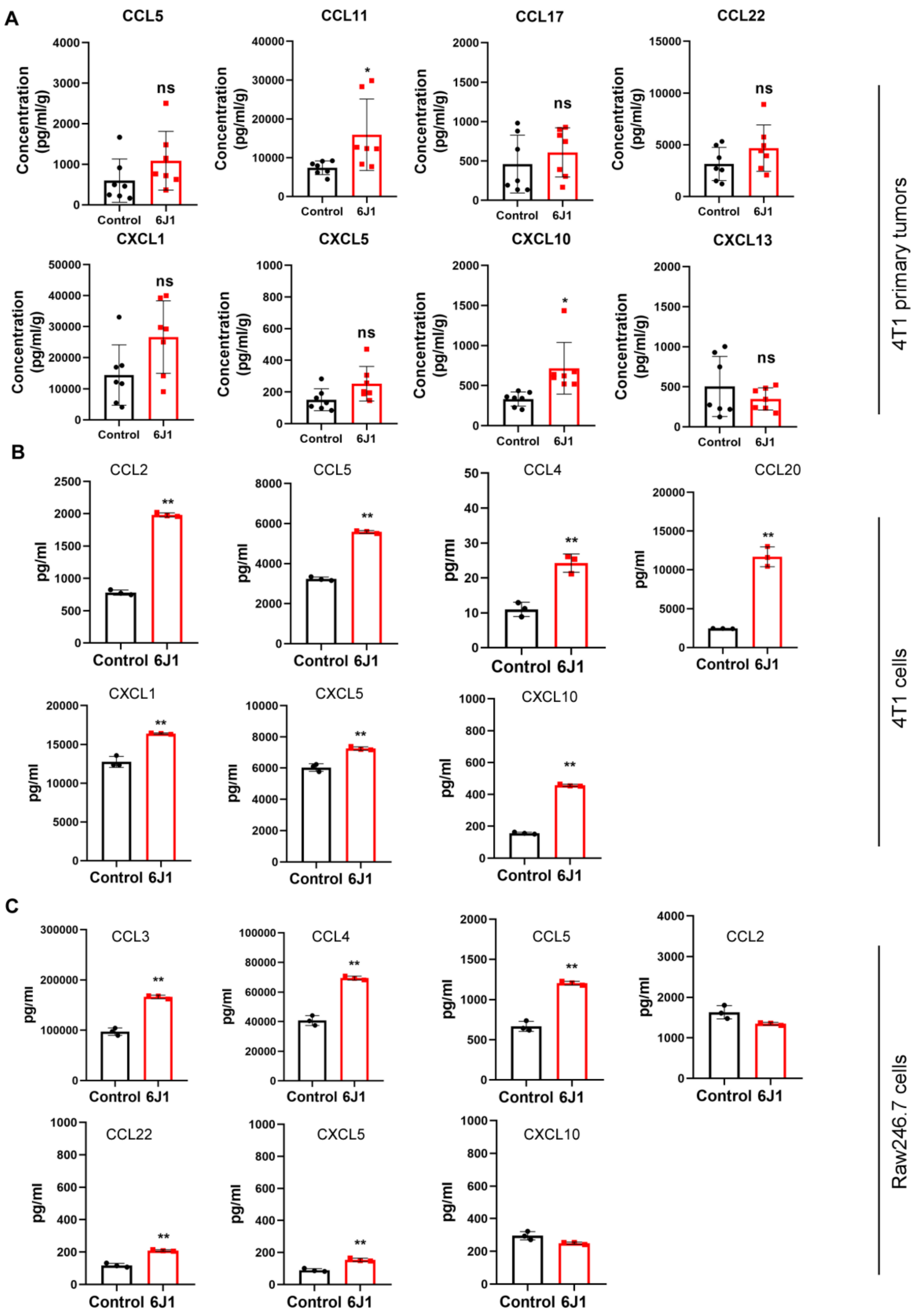

Figure S5. 6J1 induce chemokines secretion. (**A**) 4T1 cells were injected into the fat pads of female Balb/c mice, and treated with either buffer or 6J1 (30 mg/kg, daily) via oral gavage for 4 weeks. Flow cytometry-based quantification of the chemokine level in the microenvironment of 4T1 primary tumors treated with the vehicle (control) or

6J1 (30 mg/kg) daily were performed. (**B, C**) Flow cytometry-based quantification of the chemokine level in the supernatant of 4T1 (**B**) or Raw246.7 cells (**C**) treated with or without 6J1 (1 μM). In **(A-C)**, the graphs represent the mean ± s.e.m of three independent experiments, and the asterisks indicate significant differences at *P < 0.05, ** P < 0.01 or ***P < 0.001. 'ns' indicates that data were not significantly different.

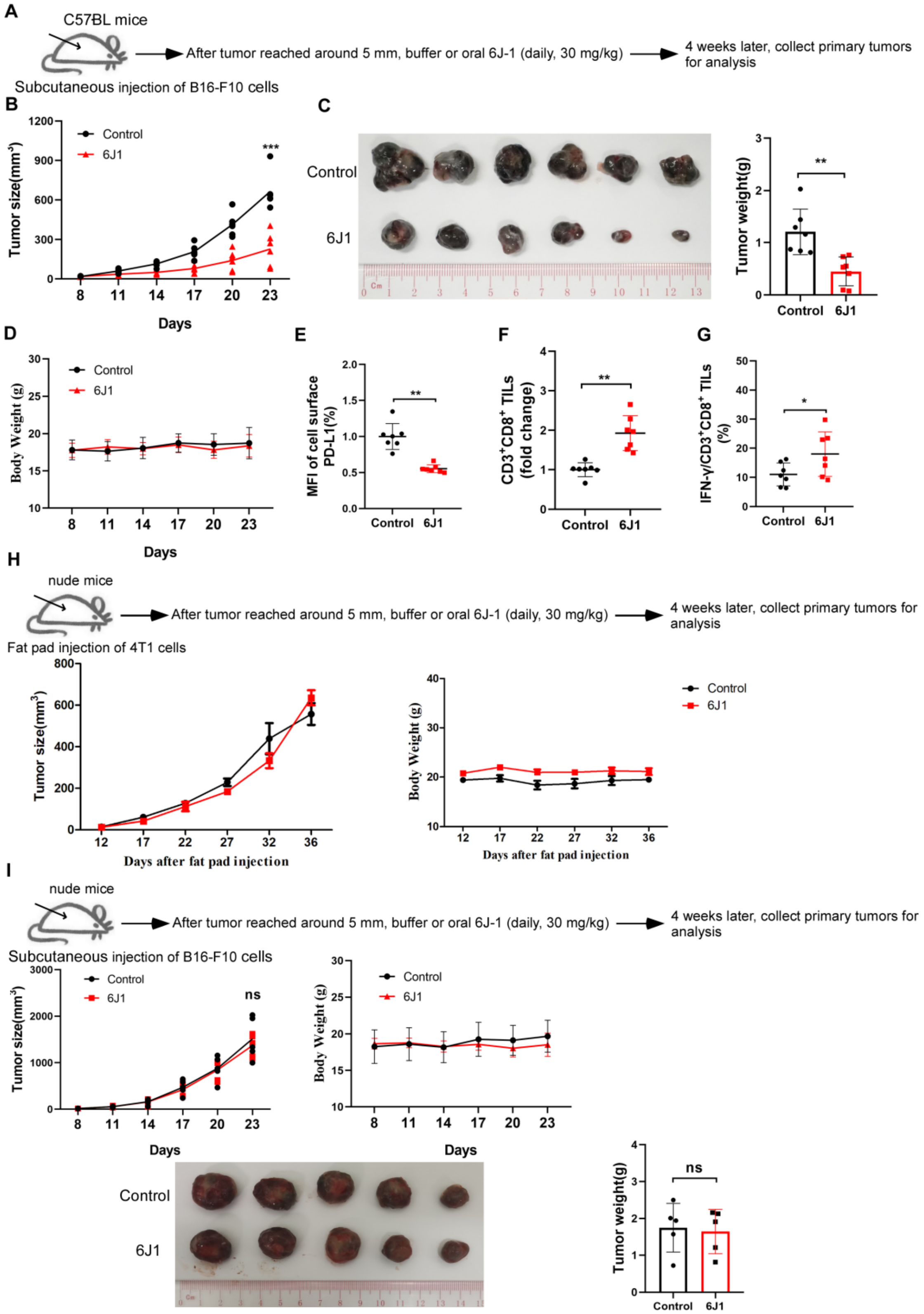

Figure S6. 6J1 suppresses the growth of primary tumors in syngeneic mouse models. (A-G) B16-F10 cells were subcutaneously injected into the flank of female C57BL/6J mice. The mice were randomly divided (n=6 per group) and treated with either buffer or 6J1 (30 mg/kg, daily, oral gavage) for 4 weeks (**A**). The tumor size **(B)**

and body weight of mice **(D)** were measured at the indicated time points. The morphology and weight of tumors at the last time point are shown **(C)**. Flow cytometry-based quantification of the levels of plasma membrane PD-L1 in the primary tumor was performed **(E)**. Fold change of CD3+CD8+ T-cell populations **(F)**, and percentage of intracellular IFN-γ **(G)** in the isolated tumor-infiltrating lymphocytes (TILs) were determined by flow cytometry analysis. **(H)** 4T1 cells were subcutaneously injected into the flank of female athymic nude mice. The mice were then randomly divided (n=4 per group) and treated with either buffer or 6J1 (30 mg/kg, daily, oral gavage). The tumor volume and body weight of the mice were measured at the indicated time points. **(I)** B16-F10 cells were subcutaneously injected into the flank of female athymic nude mice. The mice were randomly divided (n=5 per group) and treated with either buffer or V1 (30 mg/kg, daily, oral gavage). The tumor volume and body weight of mice were measured at the indicated time points. Representative images of the tumors are shown. The graphs in **(C-I)** represent the mean ± s.e.m of three independent experiments, and the asterisks indicate significant differences at *P < 0.05, ** P < 0.01, ***P < 0.001.

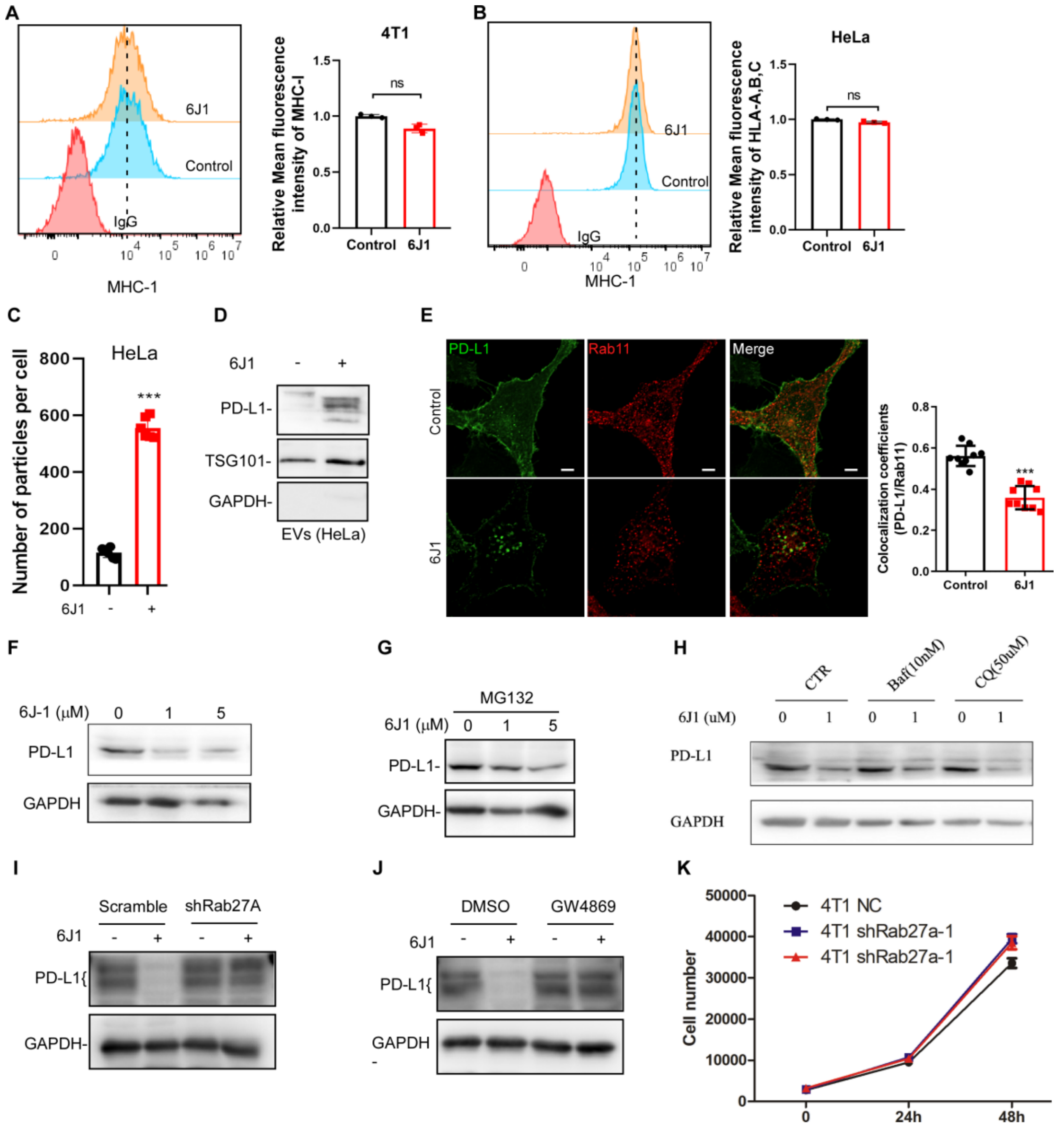

Figure S7. 6J1 induces secretion of PD-L1 via EVs. (A, B) Flow cytometry-based quantification of the plasma membrane levels of MHC-1 in 4T1 cells **(A)** or HeLa cells **(B)** treated with or without 6J1 (1 μM) for 24 h. The mean fluorescence intensity of MHC-1 was quantified. **(C, D)** HeLa cells were treated with or without 6J1 (1 μM), after which the supernatant was collected, and the concentration of extracellular vesicles (EVs) was determined using a Nanosight nanoparticle analyzer **(C)**. The EVs were then purified from the supernatant and subjected to western blot analysis **(D)**. **(E)** PD-L1-GFP-expressing HeLa cells were treated with or without 6J1 (1 μM) for 24 h, after which they were fixed and immunolabeled with the anti-Rab11 antibody (red). The colocalization coefficients (MCC) of PD-L1 with Rab11 were quantified. The scale bar is 5 μm. **(F-J)** 4T1 cells were treated with or without 6J1 for 24 h **(F)**; with or without 6J1 (1 μM) in the presence of MG132 (1 μM) for 24 h **(G)**; or with or without 6J1 (1 μM) in the presence of Bafilomycin A1 (10 nM) or CQ (50 μM) for 24 h **(H)**. In each case, the cell lysates were then subjected to western blot analysis. **(I)**

Control or Rab27a-knockdown 4T1 cells were treated with or without 6J1 (1 μM) for 24 h, and the cell lysates were then subjected to western blot analysis. (**J**) 4T1 cells in the presence or absence of GW4869 (10 μM) were treated with or without 6J1 (1 μM) for 24 h, and the cell lysates were then subjected to western blot analysis. (**K**) Proliferation of 4T1-shNC and 4T1-shRab27a cells *in vitro*. The bar graphs represent the mean ± s.e.m of three independent experiments, and the asterisks indicate significant differences at ***P < 0.001. 'ns' indicates data that were not significantly different.

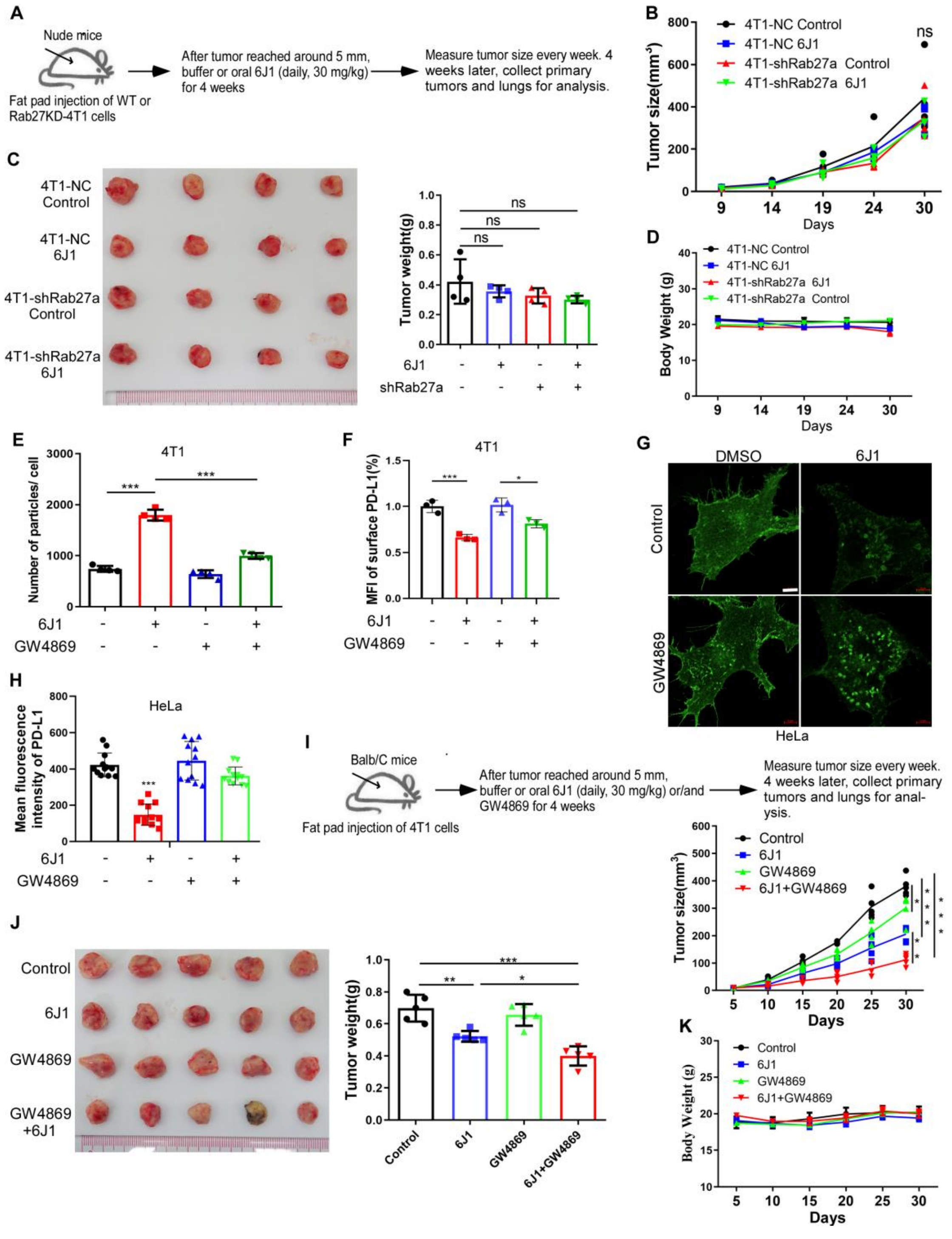

Figure S8. Blocking exosome secretion enhances the anticancer activity of 6J1. (A-D) 4T1-shNC or 4T1-shRab27a cells were injected into the fat pads of female athymic nude mice. The mice were randomly divided (n=4 per group) and treated with either buffer or 6J1 (30 mg/kg, daily) via oral gavage. The tumor size **(B)** and body weight of mice **(D)** were measured at the indicated time points. The morphology and tumor weight were recorded at the end time point **(C)**. **(E)** 4T1 cells were treated with 6J1 (1 μM) or/and GW4869 (10 μM) for 24 h, after which the supernatant was collected, and the concentration of extracellular vesicles (EVs) was determined using

the Nanosight nanoparticle analyzer. **(F)** Flow cytometry-based quantification of the levels of plasma membrane PD-L1 in 4T1 cells treated with or without 6J1 (1 μM) and/or GW4869 (10 μM) for 24 h. The mean fluorescence intensity of PD-L1 was quantified. **(G)** PD-L1-GFP expressing HeLa cells were treated with 6J1 (1 μM) and/or GW4869 (10 μM) for 24 h, after which they were fixed and imaged. The scale bar is 5 μm. **(H)** The mean fluorescence intensity of PD-L1 shown in **(G)** was quantified. **(I-K)** 4T1 cells were injected into the fat pads of female Balb/c mice. The mice were then randomly divided (n=5 per group) and treated with either buffer or V1 (30 mg/kg, daily, oral) and/or GW4869 (2.5 mg/kg, daily, IP). The tumor volume **(I)** and body weight of mice **(K)** were measured at the indicated time points. The morphology and tumor weight were recorded at the end time point **(J)**. The graphs represent the mean ± s.e.m of three independent experiments, and the asterisks indicate significant differences at *P < 0.05, ** P < 0.01 or ***P < 0.001. ‘ns’ indicates data that were not significantly different.

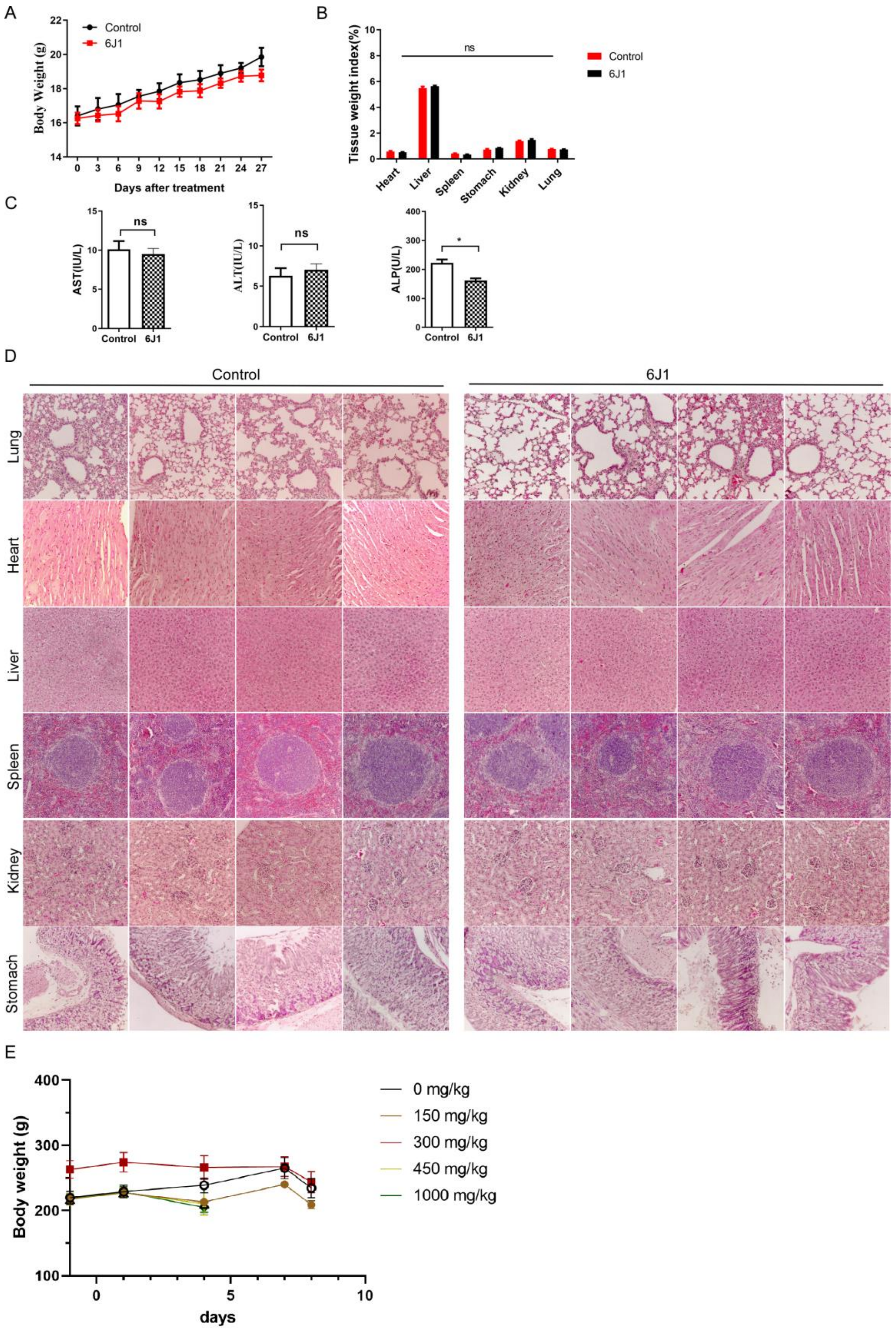

Figure S9. The subchronic toxicity of 6J1 in mice. 6 weeks old Balb/C mice were treated with vesicle control (PEG400/Ethanol/tween80, 1:1:1) or 6J1 (30 mg/kg, per day) via oral route for 4 weeks. (**A**) The body weight of mice in each group were

measured every three days during the course of drug treatment. (**B**) Tissue weight index (tissue weight/body weight x100%) were measured. (**C, D**) At the end of experiment, the blood was collected from mice in each group for AST, ALT, and ALP measurement (**C**); and the mice were then sacrificed, and the major organs were isolated, examined, and subjected to H&E staining (**D**). (**E**) Bodyweight changes of rats following oral gavage administration of 6J1 at dose levels of 0 (vehicle), 150, 450, and 1000 mg/kg once daily for up to 7 consecutive days. Note, rats of 300 mg/kg group were tested separately from other groups (showing higher initial weight than other groups), and rats of 450 and 1000 mg/kg groups were terminated on day 4 due to severe clinical findings.

Video S1. The internalization of membrane PD-L1 in PD-L1-GFP expressing HeLa cell by TIRF microscopy.

Tables. Pharmacokinetic parameters of 6J1

Table S1. Pharmacokinetic parameters of 6J1 in the plasma of male rat (15 mg/kg, oral)

Rat#	HL_Lambda_z	T_{max}	C_{max}	AUC_{last}	MRT_{last}
1	1.26	2.00	592	2844	3.56
2	1.77	0.500	630	3231	3.81
3	1.56	0.500	1000	3365	2.98
4	2.47	1.00	1930	5829	2.88
5	2.61	1.00	1450	4550	3.33
6	1.29	1.00	676	2992	3.61
Average	1.83	1.00	1046	3802	3.36
SD	0.585	0.548	541	1163	0.369
CV%	32.0	54.8	51.7	30.6	11.0

Table S2. Pharmacokinetic parameters of 6J1 in the plasma of male rat (15 mg/kg, intravenous injection)

Rat#	HL_Lambda_z	T_{max}	C_{max}	AUC_{last}	MRT_{last}
7	2.42	0.0333	10700	8603	1.68
8	1.70	0.0333	11200	8516	1.71
9	3.45	0.0333	9800	9864	2.88
10	3.16	0.0333	7460	7696	2.35
11	2.61	0.0333	11100	9254	2.21
Average	2.67	0.0333	10052	8786	2.17
SD	0.681	0.00	1551	818	0.497
CV%	25.5	0.00	15.4	9.31	23.0

Bioavailability F% $=AUC_{last\ oral}/AUC_{last\ intravenous}$ *100 = 43.3%
